# Bacterial catabolism of acetovanillone, a lignin-derived compound

**DOI:** 10.1073/pnas.2213450119

**Published:** 2022-10-18

**Authors:** Gara N. Dexter, Laura E. Navas, Jason C. Grigg, Harbir Bajwa, David J. Levy-Booth, Jie Liu, Nathan A. Louie, Seyed A. Nasseri, Soo-Kyeong Jang, Scott Renneckar, Lindsay D. Eltis, William W. Mohn

**Affiliations:** ^a^Department of Microbiology & Immunology, Life Sciences Institute and BioProducts Institute, The University of British Columbia, Vancouver, BC, V6T 1Z3, Canada;; ^b^Department of Chemistry, The University of British Columbia, Vancouver, BC, V6T 1Z3, Canada;; ^c^Department of Wood Science, Faculty of Forestry, BioProducts Institute, The University of British Columbia, Vancouver, BC, V6T 1Z4, Canada

**Keywords:** bacterial catabolism, acetovanillone, hydroxyacetophenone, lignin, *Rhodococcus*

## Abstract

Bacterial catabolic pathways have considerable potential as industrial biocatalysts for the valorization of lignin, a major component of plant-derived biomass. Here, we describe a pathway responsible for the catabolism of acetovanillone, a major component of several industrial lignin streams. *Rhodococcus rhodochrous* GD02 was previously isolated for growth on acetovanillone. A high-quality genome sequence of GD02 was generated. Transcriptomic analyses revealed a cluster of eight genes up-regulated during growth on acetovanillone and 4-hydroxyacetophenone, as well as a two-gene cluster up-regulated during growth on acetophenone. Bioinformatic analyses predicted that the hydroxyphenylethanone (Hpe) pathway proceeds via phosphorylation and carboxylation, before β-elimination yields vanillate from acetovanillone or 4-hydroxybenzoate from 4-hydroxyacetophenone. Consistent with this prediction, the kinase, HpeHI, phosphorylated acetovanillone and 4-hydroxyacetophenone. Furthermore, HpeCBA, a biotin-dependent enzyme, catalyzed the ATP-dependent carboxylation of 4-phospho-acetovanillone but not acetovanillone. The carboxylase’s specificity for 4-phospho-acetophenone (*k*_cat_/*K*_M_ = 34 ± 2 mM^−1^ s^−1^) was approximately an order of magnitude higher than for 4-phospho-acetovanillone. HpeD catalyzed the efficient dephosphorylation of the carboxylated products. GD02 grew on a preparation of pine lignin produced by oxidative catalytic fractionation, depleting all of the acetovanillone, vanillin, and vanillate. Genomic and metagenomic searches indicated that the Hpe pathway occurs in a relatively small number of bacteria. This study facilitates the design of bacterial strains for biocatalytic applications by identifying a pathway for the degradation of acetovanillone.

## Significance

Upgrading lignin, an underutilized component of biomass, is essential for sustainable biorefining. Biocatalysis has considerable potential for upgrading lignin, but our lack of knowledge of relevant enzymes and pathways has limited its application. Herein, we describe a microbial pathway that catabolizes acetovanillone, a major component of several industrial lignin streams. This pathway is unusual in that it involves phosphorylation and carboxylation before conversion to the intermediate, vanillate, which is degraded via the β-ketoadipate pathway. Importantly, the hydroxyphenylethanone catabolic pathway enables bacterial growth on softwood lignin pretreated by oxidative catalytic fractionation. Overall, these insights greatly facilitate the engineering of bacteria to biocatalytically upgrade lignin.

Lignin is a heterogeneous aromatic biopolymer that constitutes up to 35% of lignocellulosic biomass (1, 2). Due to its abundance, lignin has considerable potential as a renewable feedstock for biobased materials and chemicals. Nevertheless, it remains underutilized due to its structural heterogeneity, which presents challenges for selective depolymerization and upgrading in biorefining (1, 3). Consequently, lignin is often burned to provide heat energy for the extraction of carbohydrates. One solution to this challenge that is gaining traction is catalytic fractionation, a process that not only separates biomass into lignin and carbohydrate fractions but also promotes conversion of the lignin into aromatic monomers and oligomers (4). The monomers can be used industrially for applications, such as polymer synthesis, or can be further converted by microbial cell factories to any of a number of useful chemicals in high atom yield (5). This biocatalytic conversion harnesses the convergent nature of bacterial aromatic catabolism whereby upper pathways transform a diversity of substrates to a small number of key intermediates, typically catechols, which are further transformed to central metabolites via lower pathways (3, 6). Biocatalytic conversion depends on elucidating and optimizing the requisite aromatic catabolic pathways.

Among catalytic fractionation strategies, oxidative catalytic fractionation (OCF) offers particular promise for softwood biomass, depolymerizing the lignin to phenolic aldehydes, acids, and ketones through cleavage of intersubunit C-C linkages (7, 8). For example, Zhu et al. (7) used OCF to convert 70% of the carbon of a softwood to useful products. Of the lignin, 29% by weight was recovered as monophenolics, mainly vanillin and acetovanillone (AV), and ∼57% by weight as small oligophenolics (<600 Da), such as methoxylated biphenyls. Using a similar approach, Abdelaziz et al. (9) oxidatively depolymerized a softwood kraft lignin to a mixture of monoaromatic compounds, including vanillate, vanillin, guaiacol, and AV (9). Such mixtures lend themselves to biocatalytic conversion, as numerous bacterial strains grow on most of these compounds, with the exception of AV. Ravi et al. (10) tested nine strains of *Rhodococcus* and *Pseudomonas* for growth on depolymerized kraft lignin containing 4-hydroxybenzoate, vanillate, vanillin, guaiacol, and AV. Of these compounds, AV was the only one not degraded by any of the tested strains.

Members of the genus *Rhodococcus* are of particular interest due to their ability to degrade an exceptionally wide range of aromatic compounds and their demonstrated use at industrial scale to produce thousands of tons of acrylamide annually (11, 12). Illustrative of the aforementioned convergent catabolism, rhodococci catabolize vanillate via protocatechuate using vanillate *O*-demethylase, encoded by *vanAB;* vanillin via vanillate, using vanillin dehydrogenase (*vdh*) (13); and guaiacol via catechol, using guaiacol *O*-demethylase (*gcoAB*) (14). Protocatechuate and catechol are converted to central metabolites via a convergent β-ketoadipate pathway (15). In contrast to these well characterized pathways, a pathway for catabolism of AV has yet to be elucidated.

We recently isolated two strains of *Rhodococcus rhodochrous,* GD01 and GD02, for their ability to grow on AV (16). GD02 removed the major monoaromatic compounds, including AV, present in black liquor, an industrially relevant stream generated in the kraft pulping process. GD01 and GD02 are remarkably similar to *R. rhodochrous* EP4, a strain that was isolated for its ability to catabolize alkylphenols and alkylguaiacols produced by the reductive catalytic fractionation of biomass (14, 17). However, EP4 did not grow on AV. Genomic analyses revealed that EP4 does not encode a predicted biotin-dependent carboxylase found in GD01 and GD02. The carboxylase, annotated as HpeCBA (formerly ApkCBA), is predicted to comprise a biotin carboxyl carrier protein (HpeC), a carboxyl transferase (HpeB), and a biotin carboxylase (HpeA) (16). The predicted carboxylase shares ∼50% amino acid sequence identity with XccBCA, a three-subunit carboxylase involved in the anaerobic catabolism of a chemical analog of AV, 4-hydroxyacetophenone (HAP), by *Aromatoleum aromaticum* strain EbN1 (18). The *hpeC* gene was up-regulated ∼100-fold during growth of GD02 on a mixture of monoaromatics that included AV (16). Collectively, these results strongly implicate HpeCBA in the catabolism of AV. However, the function of HpeCBA has yet to be validated and the AV catabolic pathway has yet to be elucidated.

Herein, we describe the catabolism of alkyl-phenyl ketones by GD02. We first assembled and annotated a high-quality genome sequence of GD02. Based on the predicted catabolic pathways, we then tested the ability of this strain to grow on a variety of aromatic compounds. To identify the relevant catabolic pathways, we analyzed the transcriptomes of GD02 growing on three alkyl-phenyl ketones: AV and HAP, both of which are hydroxyphenylethanones, and acetophenone (AP), which lacks the hydroxyl group. Bioinformatic analysis of the up-regulated genes enabled us to propose a catabolic pathway for the hydroxyphenylethanones, involving phosphorylation, carboxylation, and β-elimination, as well as a separate pathway for AP, involving a Baeyer–Villiger monooxygenase. Key steps of the former pathway were validated using purified enzymes and their hypothesized substrates. Finally, we investigated the ability of GD02 to grow on monoaromatic compounds, including AV, from OCF of lodgepole pine. The results are discussed with respect to related catabolic pathways and enzymes, as well as the engineering of biocatalysts to valorize lignin.

## RESULTS

### Genome Sequence.

To better understand how GD02 catabolizes AV and related compounds, we first generated an improved genome assembly for the strain. Previously existing Illumina short-read data (16) plus new Oxford Nanopore long-read data were assembled with Unicycler as a short-read–first hybrid assembly pipeline. Overall, the hybrid assembly improved the quality of the genome sequence and increased completeness from 99.2 to 99.8%. More significantly, this closed the assembly previously comprised of 24 scaffolds, revealing three genomic elements (*SI Appendix*, Fig. S1): a circular 5,587,176-bp chromosome, a 721,409-bp linear plasmid (pRGD1), and a 165,007-bp circular plasmid (pRGD2). The new assembly is 6.47 Mb and contains 5,932 predicted genes, 147 more genes than the previous assembly.

Analysis of the annotated genome of GD02 revealed numerous genes predicted to encode aromatic catabolism (Table 1 and *SI Appendix*, Table S1), which are mainly concentrated in one region of the genome (*SI Appendix*, Fig. S1) and which were mostly previously reported (16). The genomes of GD01 and GD02 are nearly identical (average nucleotide identity, 99.1%). However, GD01 lacks pRGD2, which contains a second set of genes encoding vanillate-*O*-demethylase, *vanA2B2*. The encoded proteins VanA and VanA2 share 97.8% amino acid sequence identity, while VanB and VanB2 share 68.9% identity. Proximal to *vanA2B2* on pRGD2 are genes predicted to encode mycothiol-dependent oxidation of formaldehyde, produced in the *O*-demethylation of vanillate.

**Table 1.** Annotated hydroxyphenylethanone and AP catabolic genes of GD02

Catabolism type and genes	Locus*	Gene product	Closest homolog^†^	%ID^‡^
Hydroxyphenylethanone catabolism				
* hpeH*	*02990*	Hydroxyphenylethanone kinase, α subunit	*creH*, *Corynebacterium glutamicum,* WP_011013724.1	46
* hpeI*	*02985*	Hydroxyphenylethanone kinase, β subunit	*creI*, *C. glutamicum,* WP_011013725.1	40
* hpeC*	*02980*	Phosphophenylethanone carboxylase, biotin carboxyl carrier protein	*xccB*, *Aromatoleum aromaticum* EbN1, WP_011236025.1	51
* hpeB*	*02975*	Phosphophenylethanone carboxylase, carboxyltransferase	*xccC*, *A. aromaticum* EbN1, WP_011236024.1	51
* hpeA*	*02970*	Phosphophenylethanone carboxylase, biotin carboxylase	*xccA*, *A. aromaticum* EbN1, WP_011236023.1	45
* hpeD*	*02965*	Phosphophenyl-β-ketopropionate phosphatase	*nagD*, *Escherichia coli* K12, WP_000153129.1	24
* hpeE*	*02960*	Hydroxyphenyl-β-ketopropionyl-coA synthetase	*acsA2*, *Sinorhizobium meliloti,* WP_010968769.1	42
* hpeF*	*02955*	Hydroxyphenyl-β-ketopropionyl-coA hydrolase	*couO*, *Rhodococcus jostii* RHA1, WP_011597356.1	95
AP catabolism				
* acpB*	*09510*	Phenyl acetate esterase	*nlhH*, *Mycobacterium tuberculosis* ATCC 25618, WP_003407276.1	41
* acpA*	*09515*	Acetophenone monooxygenase	*pamO*, *Thermobifida fusca* YX, WP_011291921.1	55

ID, identity.

^*^Complete GD02 loci include the following prefix, LCH94_XXXXX.

^†^Closest homolog of experimentally verified function (gene name, strain, encoded protein identifier).

^‡^Percent amino acid sequence identity calculated over the entire length of the proteins encoded by the genes.

**Fig. 1.**
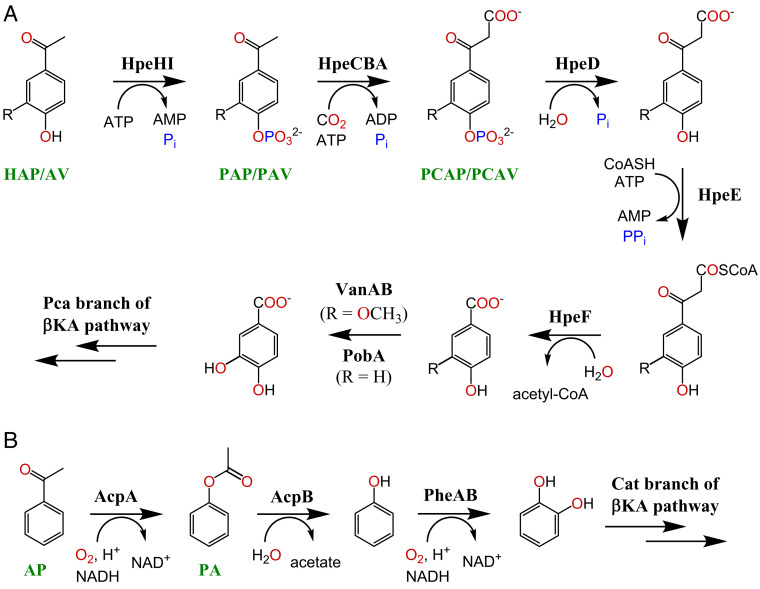
Proposed pathways for degradation of hydroxyphenylethanones (*A*) and AP (*B*). In the catabolism of HAP (R = H), HAP is phosphorylated to PAP, then carboxylated to PCAP. In the catabolism of AV (R = OCH_3_), AV is phosphorylated to PAV, then carboxylated to PCAV. Pca, protocatechuate; βKA, β-keto adipate; Cat, catechol.

We found that the *hpeCBA* genes (formerly *apkCBA*), encoding the carboxylase previously associated with AV degradation (16), were part of a cluster containing eight catabolic genes in the same orientation (*SI Appendix*, Fig. S2). Based on the following annotations, we hypothesized that this cluster encodes a pathway responsible for catabolism of hydroxyphenylethanones, including AV and HAP (Fig. 1*A*). The first two genes in this cluster were predicted to encode a kinase, HpeHI, whose closest characterized homolog, CreHI from *Corynebacterium glutamicum* (Table 1), initiates degradation of 4-cresol by phosphorylation (19). HpeHI and CreHI are homologs of phenylphosphate synthase, which initiates anaerobic phenol catabolism in *Thauera aromatica* (20), *Geobacter metallireducens* (21), and *Desulfatiglans anilini* (22). Phylogenetic analyses revealed that the kinases predicted to catalyze the phosphorylation of hydroxyphenylethanones, phenol, and 4-alkylphenols form distinct respective clades (*SI Appendix*, Figs. S3 and S4). In addition, the two components share congruent phylogenies. Based on these observations, we hypothesized that HpeHI similarly initiates AV degradation.

The next three genes encode the three-component, biotin-dependent carboxylase, HpeCBA. Because its homolog, XccBCA, is involved in the catabolism of HAP (18), we hypothesized that the Hpe pathway might degrade HAP in addition to AV. The closest characterized homolog of the product of the fifth gene, HpeD, is NagD from *Escherichia coli* K12, which catalyzes the dephosphorylation of a broad range of C5 and C6 sugars (23). Thus, we hypothesized that HpeD dephosphorylates the product of the HpeCBA reaction. The final two genes in the cluster, *hpeE* and *hpeF*, are predicted to encode an adenosine monophosphate (AMP)–forming acyl-coenzyme A (CoA) synthetase and a β-ketoacyl-CoA hydrolase, respectively. We propose that these catalyze CoA thioesterification of the carboxylated products of HpeD and the subsequent β-elimination of acetyl-CoA. Homologs of HpeE and HpeF, CouL and CouO, respectively, catalyze identical or analogous reactions in the degradation of coumarate and ferulate by various rhodococci (24). The proposed Hpe pathway produces vanillate from AV and 4-hydroxybenzoate from HAP. Vanillate is catabolized by GD02, presumably via *O-*demethylation to protocatechuate by VanAB (16). We identified a gene encoding a predicted 4-hydroxybenzoate 3-monooxygenase, PobA, which hydroxylates 4-hydroxybenzoate to protocatechuate (*SI Appendix*, Table S1).

The *hpe* gene cluster ends with a gene encoding a transposase and a duplicated fragment of *hpeF* (*SI Appendix*, Fig. S2). There is also a transposase gene immediately upstream of the cluster as well as integrase and recombinase genes downstream. Blast searches identified complete, syntenous *hpe* catabolic gene clusters in only four additional genomes, those of *R. rhodochrous* J4, *Rhodococcus* sp. JVH1, *Rhodococcus* sp. (metagenome assembled genome), and *Actinomadura macra* NBRC 14102. These genomes all contain both *pobA* and *vanAB* genes, so they might use both AV and HAP. Diverse bacteria have gene clusters containing various combinations of *hpe* gene homologs (*SI Appendix*, Fig. S5).

### Growth Experiments.

We next tested the ability of GD02 to grow on a range of aromatic substrates. GD02 was previously reported to grow on AV, guaiacol, vanillate, and vanillin (16). We confirmed growth on these substrates and identified eight additional aromatic compounds supporting growth: AP, benzoate, HAP, 2-hydroxyacetophenone, 4-hydroxybenzoate, 4-methylguaiacol, 4-methylphenol, and phenol (*SI Appendix*, Table S2). Growth on guaiacol and 4-methylguaiacol is consistent with the presence of *gcoAB* in the GD02 genome. GD02 did not grow on 4-propylguaiacol or 4-ethylphenol, which is consistent with the absence of *agcAB* and *aphAB,* respectively (14, 17). GD02 did not grow on syringate, syringaldehyde, or 4-alkyl syringols, which is consistent with the absence of genes predicted to encode the degradation of these compounds. GD02 also did not grow on two biaryl compounds that are model lignin degradation intermediates: 2,2'-dihydroxy-3,3′-dimethoxy-5,5′-dicarboxybiphenyl and guaiacylglycerol-β-guaiacyl ether. In accordance with their highly similar genomes, GD01 had the same aromatic substrate profile as GD02. However, GD02 grew on higher concentrations of AV and vanillin than did GD01: 5 mM versus 1 mM for both compounds.

### Transcriptomic Analysis.

**Fig. 2.**
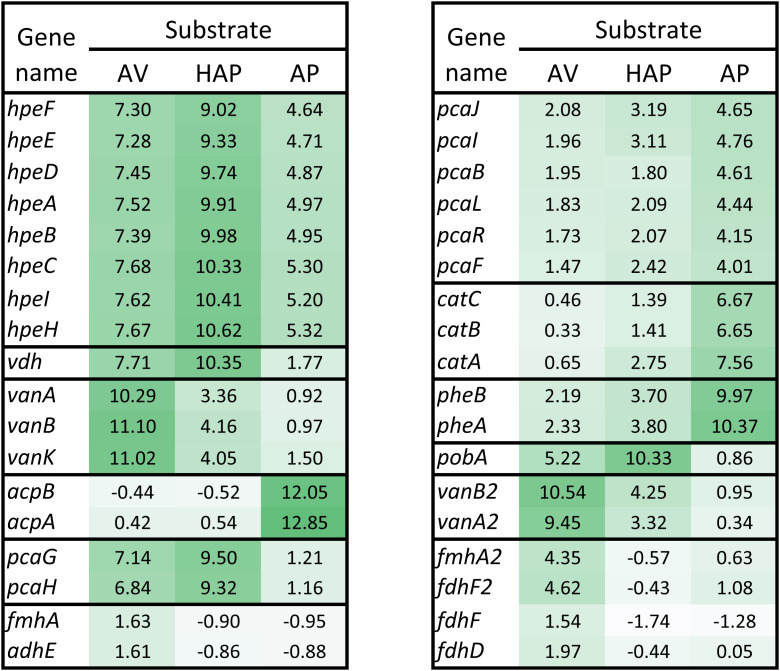
Key genes up-regulated during growth on AV, HAP, and AP. Heat map shows log2-fold increase in gene expression during growth on the aromatic substrates versus on citrate. Horizontal lines separate clusters of genes. Genes are fully identified in Table 1 and *SI Appendix*, Table S1.

To gain further insight into the basis of the ability of GD02 to grow on AV, we performed transcriptomic experiments with cells grown on AV, HAP, AP, and as a control, citrate. The *hpe* genes were among those most highly up-regulated on both AV (>150-fold) and HAP (>500-fold; Fig. 2). The *hpe* gene cluster appears to be expressed as an operon, based on similar up-regulation of the genes, cotranscription, and four-nucleotide overlaps of seven of the eight predicted genes (*SI Appendix*, Fig. S2). Two genes upstream of the *hpe* cluster (LCH94_03000 and LCH94_03005) encode putative transcriptional regulators. Although these two genes were not as highly up-regulated as the *hpe* genes, their patterns of expression on the various substrates were congruent with those of the *hpe* genes, suggesting they may be involved in regulating the putative operon. Consistent with vanillate as a catabolic intermediate of AV but not HAP, the *vanAB* and *vanA2B2* genes were highly up-regulated (>670-fold) on only AV. Interestingly, the expression pattern of *vdh*, encoding vanillin dehydrogenase, resembled that of the *hpe* genes, not *vanAB,* being most highly up-regulated on HAP. Consistent with 4-hydroxybenzoate as a catabolic intermediate of HAP but not AV, the *pobA* gene was highly up-regulated (>1,200-fold) on only HAP. The *pcaGH* genes were highly up-regulated (>110-fold) on both AV and HAP, consistent with protocatechuate as a catabolic intermediate of both substrates. However, the remaining *pca* genes, located in a separate gene cluster (*SI Appendix*, Fig. S1 and Table S1), were much less up-regulated. Read counts for the *pcaGH* and *pcaJIBLRF* genes were uniformly low on the control substrate, citrate (range, 65 to 213 average counts per gene), indicating that the latter genes were not constitutively expressed at a high level. Genes encoding mycothiol-dependent formaldehyde oxidation were also significantly up-regulated on AV.

The transcriptomic experiment indicated a distinct pathway for catabolism of AP (Fig. 1*B*). The most highly up-regulated genes on AP (>4,000-fold) were *acpAB* (Fig. 2). The *acpA* gene encodes a Baeyer–Villiger monooxygenase whose closest functionally characterized homolog is phenylacetone monooxygenase (Table 1). The *acpB* gene encodes an esterase. This suggested that AcpA oxygenates AP to form phenyl acetate (PA) and that AcpB hydrolyses PA to phenol plus acetate. Consistent with this hypothesis, GD02 grew on phenol, and the fourth and fifth most highly up-regulated genes (950-fold) on AP were *pheAB*, encoding a phenol monooxygenase that forms catechol. The genes encoding the Cat branch of the β-ketoadipate pathway, *catABC*, were also among the most up-regulated genes on AP (>100-fold), as were the *pca* genes encoding the subsequent steps of the β-ketoadipate pathway (>16-fold). Notably, none of the *acp*, *phe*, or *cat* genes was up-regulated on AV or HAP. Unexpectedly, the *hpe* gene cluster was up-regulated on AP. Blast searches revealed probable orthologs of the *acpAB* genes in diverse Actinobacteria, including six *Rhodococcus* spp. Homologs of the *acpAB* genes were also identified in diverse Proteobacteria.

### Hpe Pathway Validation.

To validate the Hpe pathway, we characterized the pathway’s predicted kinase, carboxylase, and phosphatase. The genes predicted to encode the kinase, *hpeH* and *hpeI,* and phosphatase, *hpeD,* were individually cloned and expressed in *E. coli*. HpeH, HpeI, and HpeD were purified as His-tagged proteins and the molecular masses observed in sodium dodecyl sulfate–polyacrylamide gel electrophoresis (SDS-PAGE) matched the expected masses of 71.2 kDa, 40.6 kDa, and 30.2 kDa, respectively (*SI Appendix*, Fig. S6). The three genes predicted to encode the carboxylase, *hpeCBA*, were expressed together in *Rhodococcus jostii* RHA1. HpeCBA was purified to greater than 95% apparent homogeneity using ammonium sulfate precipitation followed by anion exchange chromatography. In this preparation, the molecular masses of HpeA and HpeB as estimated by SDS-PAGE matched their calculated molecular masses of 57.4 kDa and 49.3 kDa, respectively. In contrast, the molecular mass of HpeC was estimated to be ∼28 kDa, which is considerably higher than the molecular mass of 17.4 kDa calculated from its amino acid sequence. However, a His-tagged version of HpeC, which also ran on SDS-PAGE as a band higher than 25 kDa, had a molecular mass of 19,471.4 Da according to mass spectrometry, matching its predicted molecular mass. This confirmed that the smallest band present in the HpeCBA complex corresponded to the HpeC subunit. A streptavidin-shift assay indicated that HpeC contained ∼0.7 equivalents of biotin after normalizing using the intensity of the HpeAB band (*SI Appendix*, Fig. S7). Attempts to produce HpeCBA in *E. coli* were unsuccessful. Moreover, omitting dithiothreitol and glycerol from the purification buffers led to preparations of lower specific activity.

**Fig. 3.**
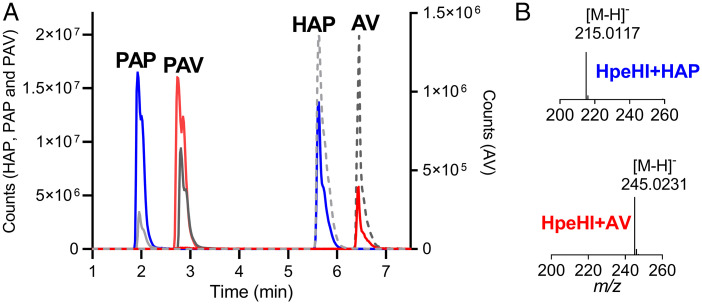
Phosphorylation of AV and HAP by HpeHI. (*A*) Extracted ion chromatograms for substrates and products of the HpeHI reaction are labeled, corresponding to both the retention time and *m/z* of standards. Reaction mixtures of HpeHI with HAP (blue) or AV (red) or no enzyme controls for HAP (dashed, light gray) or AV (dashed, dark gray) are shown. Synthesized standards for the expected products PAP (solid, light gray) and PAV (solid, dark gray) are also shown. (*B*) Mass spectra of PAP and PAV produced in the reaction (*Right*) match the expected *m/z* with <5 ppm error.

We used liquid chromatography–mass spectrometry (LC–MS) to show that HpeHI catalyzed the phosphorylation of AV and HAP to 4-phospho-acetovanillone (PAV) and 4-phospho-acetophenone (PAP), respectively. The retention times (*t*_R_s) and mass spectra of the reaction products corresponded to those of authentic standards (Fig. 3). Both protein components were required for phosphorylation. We next used a spectrophotometric assay to compare the relative ability of HpeHI to turn over the two compounds. This assay was based on the strong absorption of the phenolate ions of HAP and AV at pH 8 (*SI Appendix*, Fig. S8). In assays containing 1 μM of each kinase component, the enzyme turned over AV and HAP at rates (± SD) of 4.1 ± 0.4 min^−1^ and 3.4 ± 0.7 min^−1^, respectively.

**Fig. 4.**
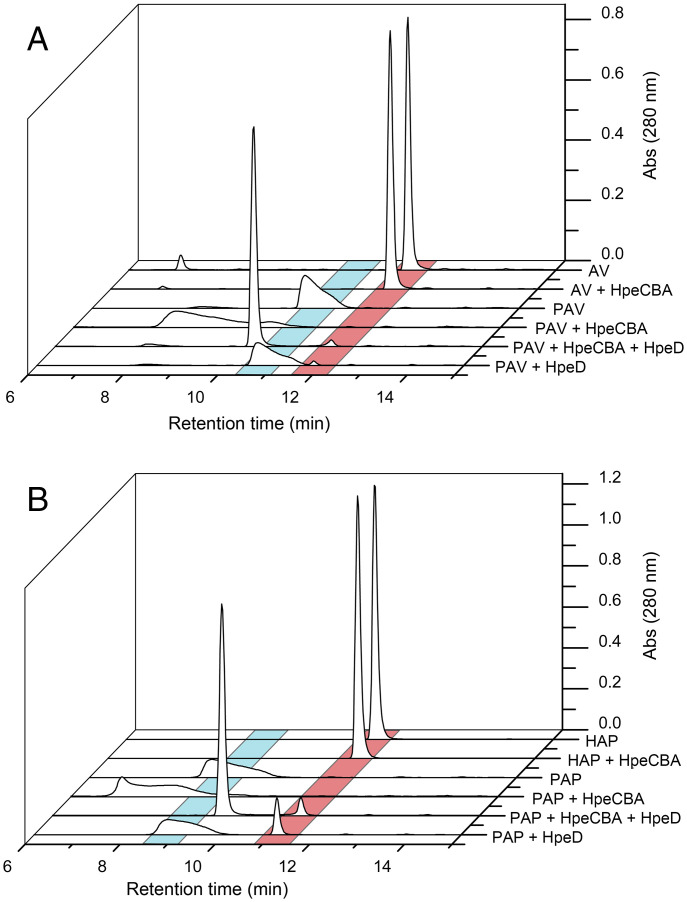
Transformations catalyzed by HpeCBA and HpeD. (*A*) AV or PAV were incubated with either HpeCBA or HpeCBA + HpeD as indicated. The product of PAV plus HpeCBA was PCAV, identified by mass spectrometry (*SI Appendix*, Fig. S9*A*). The product of PAV plus HpeCBA plus HpeD was 4-hydroxy-3-methoxyphenyl-β-ketopropionate, identified by mass spectrometry (*SI Appendix*, Fig. S9*B*). (*B*) HAP or PAP were incubated with either HpeCBA or HpeCBA plus HpeD, as indicated. The product of PAP plus HpeCBA was PCAP, identified by mass spectrometry (*SI Appendix*, Fig. S10*A*). The product of PAV plus HpeCBA plus HpeD was 4-hydroxyphenyl-β-ketopropionate, identified by mass spectrometry (*SI Appendix*, Fig. S10*B*). (*A* and *B*) Reactions were incubated for 20 min at 30 °C using 250 µM substrate in 20 mM (3-(N-morpholino)propanesulfonic acid (MOPS; *I* = 0.1 M), pH 7.5, containing 0.5 mM ATP, 4 mM MgCl_2_, and 40 mM NaHCO_3_. HPLC traces were recorded at 280 nm. Abs, absorbance.

We investigated the activities of HpeCBA in vitro using a variety of phosphorylated and unphosphorylated substrates. To this end, we synthesized PAV and PAP. High-performance liquid chromatography (HPLC) analyses indicated that reaction mixtures containing HpeCBA and PAV, depleted PAV (*t*_R_ = 10.3 min) and produced a compound with a *t*_R_ of ∼8.3 min (Fig. 4*A*). Both peaks were relatively broad. Control reactions confirmed that conversion depended on the presence of the enzyme, adenosine triphosphate (ATP), Mg^2+^, and HCO_3_^−^, as did the carboxylase activity in a coupled reaction (see below). LC–MS analysis demonstrated that the product had a mass to charge (*m/z*) value of 289.0018, corresponding to that of the predicted carboxylated product, 4-phospho-carboxyacetovanillone (PCAV; *SI Appendix*, Fig. S9*A*). Similarly, HpeCBA catalyzed the transformation of PAP to a compound with a *t*_R_ of 11.9 min (Fig. 4*B*) and *m/z* value of 259.0017, corresponding to that of the predicted carboxylated product, 4-phospho-carboxyacetophenone (PCAP; *SI Appendix*, Fig. S10*A*). Finally, under these reaction conditions, HpeCBA did not detectably transform either AV or HAP, indicating that phosphorylation is required for carboxylation of these compounds.

To investigate the activity of HpeD, we added the enzyme to the above-described carboxylation reactions. When PAV was incubated in the presence of HpeD and HpeCBA, an HPLC peak corresponding to PCAV was not detected (Fig. 4*A*). Instead, we observed a compound with a *t*_R_ of 10.1 min and an *m/z* value of 211.0613, corresponding to that of 4-hydroxy-3-methoxyphenyl-β-ketopropionate (*SI Appendix*, Fig. S9*B*). In addition, a small peak corresponding to AV was observed. Incubation of PAV with HpeD alone yielded similarly small amounts of AV. Overall, these results indicate that HpeD is a phosphohydrolase that catalyzes the conversion of PCAV to 4-hydroxy-3-methoxyphenyl-β-ketopropionate. The results further indicate that the enzyme can catalyze the hydrolysis of PAV but that its specific activity for PCAV is much higher. Finally, similar results were obtained using PAP (Fig. 4*B*, *SI Appendix*, Fig. S10*B*). That is, HpeD catalyzed the hydrolysis of PCAP to 4-hydroxyphenyl-β-ketopropionate and, at a much lower rate, PAP to HAP. Overall, these results consistently support the proposed Hpe pathway (Fig. 1*A*).

### Kinetic Properties of HpeCBA Carboxylase.

To gain insight into the overall energetics and preferred substrate of the Hpe pathway, we further characterized the novel HpeCBA carboxylase with respect to its utilization of ATP and substrate specificity. HPLC analysis of HpeCBA reactions performed using PAV and ATP revealed that adenosine diphosphate (ADP) was exclusively produced: no AMP was detected (*SI Appendix*, Fig. S11*A*). To determine the stoichiometry of ADP production, we used a continuous spectrophotometric assay in which ADP production was coupled to NADH oxidation using pyruvate kinase and lactate dehydrogenase (25). In assays containing limiting amounts of PAV, 0.93 ± 0.05 mol NADH was oxidized per mole of PAV consumed (*SI Appendix*, Fig. S11*B*). This indicates that HpeCBA catalyzes the hydrolysis of 1 mol of ATP to ADP per mole of PAV carboxylated.

The substrate specificity of HpeCBA was evaluated using the aforementioned spectrophotometric assay. Under the described conditions, the rate of NADH oxidation was directly proportional to the concentration of HpeCBA. The reaction was dependent on the presence of HCO_3_^−^, ATP, and Mg^2+^, and the pH optimum was 7.5 (*SI Appendix*, Fig. S12). This pH was used for additional steady-state kinetic studies. HpeCBA obeyed Michaelis–Menten kinetics in transforming both PAV and PAP (*SI Appendix*, Fig. S13). The enzyme’s specificity (*k*_cat_/*K*_M_) for PAP was an order of magnitude higher than for PAV (Table 2), indicating a strong preference for the former substrate. Nevertheless, the *k*_cat_ of the carboxylase for the two substrates is remarkably similar.

**Table 2.** Steady-state kinetic parameters of HpeCBA*

Substrate	*k*_cat_, s^-1^^†^	*K*_M_, μM	*k*_cat_/*K*_M_, mM^−1^ s^−1^
PAV	2.25 (0.06)	560 (30)	3.9 (0.1)
PAP	2.40 (0.07)	69 (6)	34 (2)

^*^Experiments were performed in 20 mM MOPS (*I* = 0.1 M), pH 7.5, at 25 °C.

^†^*k*_cat_ values are based on protein concentrations and may be ∼25% higher based on the biotin content of HpeC (*SI Appendix*, Fig. S7). SD of triplicates is shown in parentheses.

Finally, we tested whether the phosphatase facilitates the activity of the carboxylase, for example by generating a phenolate anion that serves as the substrate of the carboxylase. When added at a 1:1 molar ratio with respect to HpeCBA, HpeD did not detectably affect the specific activity of the carboxylase, 1.42 ± 0.05 U/mg with HpeD versus 1.42 ± 0.04 U/mg without HpeD (*SI Appendix*, Fig. S14). This indicates that HpeD does not generate a substrate for HpeCBA or interact with the carboxylase to accelerate the reaction. This result is consistent with the pH profile of HpeCBA (*SI Appendix*, Fig. S12*B*), whose specific activity declined above pH 7.5 despite the fact that the concentration of phenolate anion increases above this pH value. It is further noted that under these reaction conditions, no HpeD-catalyzed dephosphorylation of PAP was detected (*SI Appendix*, Fig. S14), indicating that the phosphatase has a much stronger substrate preference for the carboxylated product. Interestingly, the carboxylase progress curves remained linear for longer in the presence of HpeD, indicating that HpeCBA might be subject to product inhibition.

### Growth of GD02 on Pine OCF Extracts.

To investigate the potential of GD02 to transform an industrially relevant lignin stream, the strain was tested for growth on an extract of softwood (namely, lodgepole pine) biomass treated by OCF. The slurry resulting from OCF was acidified and extracted with ethyl acetate. Extraction yielded up to 15.0 ± 0.2 mM monoaromatic compounds, predominantly vanillin, AV, and vanillic acid present in a ∼5:1:1 molar ratio (*SI Appendix*, Table S3), which represented 75% of the monoaromatic compounds present in the slurry and indicated 33.9% conversion of lignin into monoaromatic compounds. The recovery of monoaromatics from OCF by ethyl acetate extraction differed based upon conditions (solvent ratios) and process (repeated extraction of liquor). OCF extracts additionally contained small organic acids, including fumaric, 2-hydroxybutyric, glycolic, and lactic acids. For growth studies, the extract was evaporated, and the residue was suspended in dimethyl sulfoxide, which does not support growth of GD02 (*SI Appendix*, Table S2). The dimethyl sulfoxide solution was added to liquid medium to yield a final concentration of up to 3 mM total aromatic compounds.

**Fig. 5.**
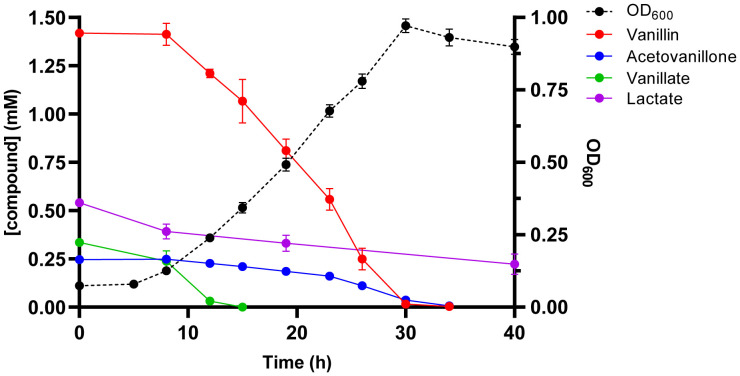
Growth of GD02 on lodgepole pine OCF extracts. GD02 was grown at 30 °C in M9+minerals containing OCF extracts at a concentration of 2 mM total aromatic compounds. Depletion of aromatic compounds from culture supernatants was measured using HPLC. Data points represent the average of triplicate experiments and the error bars represent the SD.

GD02 grew on liquid minimal medium supplemented with OCF extract up to a concentration of 2 mM total aromatic compounds. The doubling time of the bacterium was ∼9 h (Fig. 5), which was faster than on 2 mM vanillin (∼15 h) but slower than on 2 mM vanillate (∼3.3 h). HPLC analysis revealed that the three major monoaromatic compounds were efficiently removed, with vanillate depleted well before vanillin and AV. Among the small organic acids, GD02 removed 59% of the lactate but did not detectably deplete the others. GD02 did not grow on higher concentrations of OCF extract (*SI Appendix*, Fig. S15), and further experiments revealed that extract at a concentration of 3 mM total aromatics inhibited growth of GD02 on rich medium.

## Discussion

This study provides a consistent body of evidence supporting the proposed pathway for the catabolism of hydroxyphenylethanones (Fig. 1*A*). Transcriptomic analysis revealed that the *hpe* genes plus additional genes predicted to encode AV and HAP catabolism were among the most highly up-regulated genes during growth on these substrates (Fig. 2). The expected intermediate of AV catabolism, vanillate, supported growth of GD02, as did the expected intermediate of HAP catabolism, 4-hydroxybenzoate (*SI Appendix*, Table S2). The kinase (HpeHI), carboxylase (HpeCBA), and phosphatase (HpeD) were all shown to have their predicted activities on AV, HAP, and their respective intermediates (Figs. 3 and 4). Importantly, HpeCBA carboxylated only the phosphorylated intermediates, PAV and PAP, and not AV or HAP. Thus, the evidence that AV and HAP are catabolized via the Hpe pathway as proposed is strong. It is possible that AcpA might have some ability to transform AV or HAP. However, this appears unlikely, as *R. jostii* RHA1 also has *acpAB* and is able to grow on AP, but it is not able to grow on AV or HAP.

HpeHI appears to belong to a group of kinases involved in aromatic catabolism (*SI Appendix*, Figs. S3 and S4). The rationale for the energetically costly phosphorylation reaction in the Hpe pathway is unclear. One possibility is that phosphorylation transforms the hydrophobic substrate, maintaining a concentration gradient favorable for its uptake via passive diffusion while simultaneously preventing its loss from the cell via passive diffusion. Additional or alternative possibilities are that phosphorylation detoxifies the substrate or phosphorylation permits the use of a carboxylase that consumes one ATP, rather than two, per reaction.

An unusual feature of the Hpe pathway is carboxylation of the acetyl substituent of AV and HAP. Carboxylation is a common strategy in the assimilation of compounds that lack a functionalizable terminal carbon group (26). Also, several anaerobic aromatic degradation pathways utilize carboxylation. Following the phosphorylation of phenol by *T. aromatica,* 4-phenylphosphate is subsequently carboxylated by an enzyme not homologous to HpeCBA, with concomitant release of the phosphate group, forming 4-hydroxybenzoate (27). *A. aromaticum* EbN1 uses an ATP-dependent carboxylase not homologous to HpeCBA for anaerobic degradation of ethylbenzene and AP (28). As noted in the introductory text, XccBCA, a homolog of HpeCBA, is used by EbN1 for anaerobic degradation of 1-ethylphenol (18). Interestingly, the genes encoding XccBCA are clustered with homologs of *hpeHI* (*SI Appendix*, Fig. S5). Thus, while the substrate of XccBCA was not previously proposed to be phosphorylated, this may be the case. We are not aware of any aerobic aromatic degradation pathways, other than the Hpe pathway, that involve carboxylation. Notably, the catabolic steps encoded by the *hpe* genes do not require O_2_, which means the pathway may have evolved in an anaerobic organism.

GD02 appears to have acquired the Hpe pathway via horizontal gene transfer. This is consistent with the absence of the *hpe* genes in the genomes of most *Rhodococcus* strains. The presence of transposase and integrase genes upstream and downstream of the *hpe* gene cluster (*SI Appendix*, Fig. S2) is also consistent with horizontal transfer. Since the *hpe* genes are contiguous and cotranscribed, it appears that the complete gene cluster transferred. Since HAP is the preferred substrate for HpeCBA, it may be that the ability of GD02 to grow on AV is a fortuitous consequence of the cooccurrence of the Hpe pathway and the vanillate pathway.

It is surprising that the *hpe* genes are substantially up-regulated on AP (Fig. 2). Since the Hpe pathway requires a hydroxyl group on the aromatic ring, it cannot transform AP. Thus, it seems likely that AP coincidentally functions as an effector for transcriptional regulation of the *hpe* genes. Interestingly, on the substrates tested, the expression pattern of *vdh*, encoding vanillin dehydrogenase, closely resembled that of the *hpe* genes, despite the fact that Vdh is not required for catabolism of AV or HAP. Possibly, the *hpe* and *vdh* genes are coregulated as an adaptation to environments where vanillin cooccurs with hydroxyphenylethanones.

We have identified genes and proteins likely responsible for a pathway for AP catabolism, which was first proposed in 1975 (29). The previous study reported enzymes in cell-free extracts that oxidized AP to PA and hydrolyzed PA to phenol plus acetate. Enzymological studies (30) reported transformation of AP and some chemical analogs by Baeyer–Villiger monooxygenases but did not link this transformation to growth on AP. It would be informative to express and characterize acetophenone monooxygenase to confirm the Acp pathway and determine the substrate range of the enzyme. The initial steps catalyzed by AcpA and AcpB are analogous to those proposed for catabolism of HAP by *Pseudomonas fluorescens* ACB (31, 32).

The transcriptomic results indicate that AV, HAP, and AP are all funneled into the branched β-ketoadipate pathway, with AV and HAP entering via protocatechuate and AP entering via catechol. Accordingly, the *pcaGH* genes, encoding protocatechuate 3,4-dioxygenase, were highly up-regulated on both AV and HAP but not on AP (Fig. 2). Surprisingly, the *pca* genes encoding the remainder of the β-ketoadipate pathway, located in a separate cluster, were much less up-regulated on AV and HAP. It appears that the latter genes are less highly expressed than the former on AV and HAP, since they also did not have a higher constitutive level of expression. In most *Rhodococcus* strains, other than GD02, the *pca* genes occur in a single cluster (12).

Strain GD02 has metabolic capacities with strong potential for use in biocatalytic valorization of lignin. The Hpe pathway converges with the vanillin pathway, facilitating metabolism of vanillin, vanillate, and AV, the three main monoaromatic products of OCF. Accordingly, GD02 completely degraded these three components of pine OCF (Fig. 5). In a previous study, we showed that GD02 removes these three compounds from black liquor (16). These convergent pathways comprise an attractive metabolic module potentially useful for developing biocatalysts to valorize lignin treated by OCF or to valorize black liquor. Additionally, the plasmid pRGD2 encodes two processes that may protect GD02 from toxicity associated with growth on vanillin, vanillate, and AV. This protective effect is suggested by the ability of GD02 to grow on much higher concentrations of AV or vanillin than does GD01, which lacks the plasmid. The extra copy of *vanAB* genes on pRGD2 may reduce intracellular accumulation of toxic vanillin and potentially toxic AV, or Hpe pathway intermediates. Additionally, oxidation of formaldehyde encoded on pRGD2 may reduce toxicity of this product from *O*-demethylation of vanillate, occurring during degradation of vanillin, vanillate, and AV. This detoxication was previously proposed to facilitate growth of *R. jostii* RHA1 on *p*-hydroxycinnamates (24).

Higuchi et al. (33) recently described a pathway used by Sphingobium sp. SYK-6 to catabolize AV and acetosyringone. The SYK-6 pathway is encoded by the acvABCDEF genes, which are homologous and syntenous to hpeHICBAD (*SI Appendix*, Fig. S5). While we did not test it, growth of GD02 on acetosyringone is unlikely given the inability of GD02 to grow on syringate, an intermediate in acetosyringone degradation by SYK-6. Importantly, Higuchi et al. demonstrated that the acv genes are essential for growth of SYK-6 on AV, and that they conferred the ability to grow on AV when heterologously expressed in *Pseudomonas* sp. NGC7. Interestingly, SYK-6 lacks homologs of HpeE and HpeF and appears to use the functionally analogous, but non-homologous, enzymes, VceA and VceB, which were previously shown to transform hydroxyphenyl-β-ketopropionates to hydroxybenzoates (34).

## Materials and Methods

Further details of all materials and methods are provided in the *SI Appendix*.

### Chemicals and Reagents.

All reagents were purchased from Sigma-Aldrich Co. and were of analytical grade unless otherwise noted. PAP and PAV were synthesized by a published method (35) with modifications, as described in the *SI Appendix*. Enzymes for cloning were purchased from New England Biolabs. Oligonucleotides were ordered from Integrated DNA Technologies.

### Genome Sequencing, Assembly, and Annotation.

Genomic DNA for long-read sequencing was extracted from GD02 using the Genomic-tip 500/G kit (Qiagen) according to the manufacturer’s protocol, with modifications. Oxford Nanopore sequencing was performed by the Microbial Genome Sequencing Center. Long and short reads were assembled with Unicycler as a short-read first-assembly pipeline (36). The genome was annotated with the NCBI Prokaryotic Genome Annotation Pipeline.

### Growth Experiments.

*R. rhodochrous* GD01 and GD02 were grown on M9 medium (37) amended with an HCl-solubilized mineral solution (M9+minerals) described previously (38) plus organic growth substrates. The optical density at a wavelength of 600 nm (OD_600_) was monitored spectrophotometrically approximately every 24 h. Protein was quantified using the Micro BCA Protein Assay (Thermo Fisher Scientific Inc.) and a VersaMax microplate reader (Molecular Devices). Colony-forming units were determined on Luria–Bertani plates. Substrate depletion was determined by gas chromatography–mass spectrometry (GC-MS).

### Transcriptomic Experiment.

GD02 was grown on M9+minerals plus AV, HAP, AP, or citrate, and cells were harvested during midlog phase. RNA was extracted using TRIzol (Thermo-Fisher) and the Qiagen RNeasy mini kit (catalog 74104). Complementary DNA was generated with Turbo DNase (Thermo-Fisher). RNA sequencing was performed by the Sequencing and Bioinformatics Consortium at The University of British Columbia.

### Protein Production and Enzyme Assays.

HpeH, HpeI, and HpeD were produced by heterologous expression of *hpeH*, *hpeI*, and *hpeD* in *E. coli* BL-21 λ (DE3). HpeC and HpeCBA were produced by heterologous expression of *hpeC* and *hpeCBA* in *R. jostii* RHA1. HpeH, HpeI, HpeC, and HpeD were produced with N-terminal His tags and were purified using immobilized metal-affinity chromatography. The HpeCBA complex was precipitated with ammonium sulfate and purified using anion-exchange chromatography. Substrate removal and product formation by enzymes were determined by HPLC–mass spectrometry. The activity of HpeHI was evaluated spectrophotometrically based on the difference in absorbance between the substrates and phosphorylated products. Steady-state kinetic assays of HpeCBA were performed spectrophotometrically using a coupled assay and monitoring NADH oxidation.

### OCF Extract Transformation Experiments.

Lodgepole pine, *Pinus contorta*, was subjected to OCF according to the method of Zhu et al. (7). Low-molecular-weight compounds were extracted with ethyl acetate, the solvent was evaporated under N_2_, and the extracted compounds were dissolved in dimethyl sulfoxide. GD02 was grown on M9+minerals supplemented with OCF extract. Growth was monitored at OD_600_, and removal of compounds in the extract was measured by GC–MS and HPLC.

### Note added in proof.

A paper by Higuchi et al. (33) describing catabolism of AV and acetosyringone by Sphingobium sp. SYK-6 was published while the current manuscript was in review.

## Supplementary Material

Supplementary File

## Data Availability

DNA and RNA data have been deposited with the National Center for Biotechnology Information, accession numbers JAHRXG000000000 and PRJNA742134, respectively (39, 40). All study data are included in the article and/or supporting information.
