# Supplementary material for: Bacterial catabolism of acetovanillone, a lignin-derived compound
Source: Proc Natl Acad Sci U S A. 2022 Oct 18;119(43):e2213450119. doi: 10.1073/pnas.2213450119 (PMC9618137; doi:10.1073/pnas.2213450119)
Supplement: Supplementary File [file pnas.2213450119.sapp.pdf]

Supplementary Information for

Bacterial catabolism of acetovanillone, a lignin-derived compound

Gara N. Dexter, Laura E. Navas, Jason C. Grigg, Harbir Bajwa, David J. Levy-Booth, Jie Liu, Nathan A. Louie, Seyed A. Nasseri, Soo-Kyeong Jang, Scott Renneckar, Lindsay D. Eltis, and William W. Mohn

Corresponding authors:
William W. Mohn, wmohn@mail.ubc.ca; Lindsay D. Eltis, leltis@mail.ubc.ca

This PDF file includes:

Supplementary text
Figures S1 to S15
Tables S1 to S4
SI References

Other supplementary materials for this manuscript include the following:

none

Supplementary Information Text

SI Materials and Methods

Genome sequencing, assembly, and annotation – A 50-ml culture of GD02 was grown in M9 medium[1] amended with a mineral solution[2] plus 5 mM citric acid overnight to a final OD_{600} of 1.47. Genomic DNA was extracted from using Genomic-tip 500/G kit (Qiagen, Germany) according to the manufacturer's protocol with the following modifications. The culture was divided into two aliquots and centrifuged at 4000 RCF for 5 min. The supernatant was discarded, and the pellets were each suspended by vortexing in 11 mL of Buffer B1 with RNase A (Qiagen, Lot No. 8850001044). To each suspension, 300 µl of lysozyme stock (100 mg mL^{-1}, Sigma) and 500 µl of Proteinase K Solution (Invitrogen) were added, and they were incubated at 37 ˚C for 2 hours, with vortexing every 30 min. To each lysate, 4 mL of Buffer B2 were added and mixed by inversion and vortexing for a few seconds. The lysates were incubated at 50 ˚C overnight (approximately 18 h), at which time they were clear. DNA was quantified by Qubit, and 1 µg of each sample was run on a 1% agarose gel to verify size and integrity.

Short read elimination was performed using Circulomics Short Read Eliminator Kit (Lot No. 10033) according to the manufacturer's protocols, with the addition of two 2-min centrifuge steps after washing the pellets in 70% ethanol. Concentration was determined by Qubit and each sample was run on a 0.5% agarose gel along with the original sample.

Adapter and quality trimming was performed for previously generated Illumina short reads using BbDuk, and for Oxford Nanopore Technology long reads, using Porechop[3,4]. Trimmed and filtered long and short reads were used to produce a hybrid assembly using Unicycler as a short-read first assembly pipeline[5]. BUSCO and CheckM[6,7] were used to assess the quality and completeness of this assembly as well as the short read only genome assemblies of GD01 and GD02 and three other *Rhodococcus* genomes. Average nucleotide identity (ANI) was calculated using FastANI[8]. Annotation of the genome was performed using the NCBI Prokaryotic Genome Annotation Pipeline (PGAP). Aromatic degradation genes were identified by BLASTp comparison to reference amino acid sequences.

Phylogenetic analysis – Amino acid sequences were aligned using MUSCLE[9]. The phylogenetic tree was generated using the Maximum Likelihood method in MEGA X software[10] computing distances based on the JTT matrix[11], with 1000 bootstraps iterations.

Growth Experiments – GD02 cultures were tested for growth on a variety of aromatic compounds. Starter cultures were grown overnight in LB broth, pelleted at room temperature at 4000 RCF and washed with M9. The pellets were suspended in M9+minerals to an optical density (OD_{600}) of 1-1.2. A. Tested compounds were solubilized in the appropriate volume of M9+minerals to achieve a final concentration of either 1 mM or 0.5 mM and filter sterilized. A 1% inoculum was added to each 50-ml culture and flasks were grown in triplicate for each condition shaking at 200 rpm at 37 ˚C. Each experiment included triplicate no-carbon negative controls and positive controls of 1 mM citrate. Each experiment was run for a maximum of 120 h.

Substrate depletion was monitored using an Agilent Technologies (Santa Clara, U.S.A.) 6890N gas chromatograph equipped with a 30-m Agilent 190915-433 capillary column and a 5973 mass-selective detector (GC/MS). 300 µl of culture was spiked with 3-chlorobenzoic acid as an internal standard, acidified with 1% acetic acid and then extracted 1:1 (v/v) with ethyl acetate and dried under nitrogen. Samples were then derivatized with equal volumes of pyridine and trimethylsilyl (TMS).

Transcriptomic analyses – GD02 was grown in triplicate on M9+minerals with 1 mM organic substrate (AV, HAP, AP, or citrate) with shaking at 200 rpm at 37 °C to mid-log phase. Extracted RNA was run on a 1% (w/v) agarose gel and its concentration was determined by Qubit (Thermo-Fisher). Ribodepletion, library preparation (Nextera) and sequencing (NextSeq, 2x150) were performed by the Sequencing and Bioinformatics Consortium at The University of British Columbia. Transcripts were quantified using Salmon 0.8.1. Differential expression was analyzed using DeSeq2 1.18.1 in R 3.4.4.

DNA manipulation – DNA was propagated, purified and manipulated using standard protocols[1]. *E. coli* and RHA1 were transformed with DNA by electroporation using a MicroPulser with GenePulser cuvettes (Bio-Rad). The expression vector pTip-*hpeCBA* and pTip-*hpeC* were constructed by amplifying *hpeCBA* and *hpeC* as single fragments from GD02 genomic DNA using *hpeCBA*-For and *hpeCBA*-Rev for *hpeCBA* and *hpe*C-For and *hpeC*-Rev for *hpeC* (Table S4). The resulting amplicons were inserted into *Nde*I/*Hind*III-linearized pTipQC2 using T4 ligase. The pET-*hpeD* vector was constructed by amplifying *hpeD* using *hpeD*-For and *hpeD*-Rev (Table S4). The resulting amplicon was cloned into pET15b that had been linearized using *Nde*I/*Hind*III using Gibson Assembly. The nucleotide sequences of the constructs were verified (GENEWIZ). Water for buffers was purified using a Barnstead Nanopure Diamond™ system to a resistivity of 18 MΩ.

HpeH, HpeI and HpeD production and purification – HpeH, HpeI and HpeD were produced heterologously as N-terminal polyHis-tagged (Ht-) proteins using *E. coli* BL-21 λ (DE3) containing pET-*hpeH,* pET-*hpeI,* or pET-*hpeD*. Freshly transformed cells were grown with shaking at 200 rpm at 37 °C in LB supplemented with 50 mg L^{-1} of ampicillin, until the culture reached an OD_{600} of ~0.6. Expression was induced with 0.5 mM isopropyl β-D-thiogalactopyranoside, and the cells were incubated at 30 °C for an additional 16 h. Cells from 1 L of culture were pelleted by centrifugation at 4 °C and resuspended in 20 mL of lysis buffer containing 50 mM sodium phosphate, 300 mM NaCl, 10 mM imidazole (omitted for HpeH and HpeI), pH 8.0, 2 tablets of proteinase inhibitor (cOmplete™, Mini) and DNAseI (2 µg mL^{-1}). Cells were lysed at 4 °C using an EmulsiFlex-C5 homogenizer (Avestin). Cellular debris was removed by centrifugation and the soluble portion was filtered (0.45 µm). Proteins were purified from the cell extract using immobilized metal affinity chromatography (Ni-NTA, Thermo Fisher Scientific, Waltham, MA, USA) according to the manufacturer's protocol. The fractions containing HpeH, HpeI, or HpeD, as judged by SDS-PAGE, were pooled and dialyzed overnight against 50 mM sodium phosphate, 150 mM NaCl, pH 8.0. Protein preparations were concentrated, flash frozen in liquid N_2, and stored at -80 °C until further use.

HpeCBA production and purification – RHA1 freshly transformed with pTip-*hpeCBA* was grown in LB with shaking at 200 rpm at 30 ˚C. This culture was used to inoculate 1 L of LB supplemented with 34 µg mL^{-1} chloramphenicol and 20 µg mL^{-1} of biotin and

grown until it reached an OD_{600} of ~0.8. Expression of *hpeCBA* was induced with 5 μg mL^{-1} thiostrepton, and the cells were incubated for an additional 24 h. Cells were harvested by centrifugation. Cells collected from 1 L of culture were suspended in 20 mL of buffer A (20 mM MOPS, I = 0.1 M, pH 7.5) containing 2 tablets of proteinase inhibitor (cOmplete™, Mini). The cell suspension was subjected to five rounds of bead-beating at 6 m/s using a FastPrep®-24 (MP Biomedicals) with 5 min on ice between rounds. Cellular debris was removed by centrifugation (40,000 RCF for 40 min) and ammonium sulfate was added to the supernatant to a final concentration of 1.3 M followed by another round of centrifugation. The supernatant was removed. The pellet of precipitated proteins containing HpeCBA was suspended in buffer A supplemented with 2 mM DTT and 10% glycerol, and dialyzed overnight against the same buffer to remove residual ammonium sulfate. The protein preparation was loaded onto a MonoQ 10/100 GL column (GE Healthcare) run using an ÄKTA Purifier. Proteins were eluted with a linear gradient of buffer B (buffer A + 2 mM DTT + 10% glycerol + 1 M NaCl). HpeCBA-containing fractions, as judge by SDS-PAGE, were pooled and concentrated to ~5 mg mL^{-1} using an Amicon Ultra-15 centrifugal filtration unit (Millipore) equipped with a 30 kDa cut-off membrane, flash frozen in liquid N_2, and stored at -80 °C.

HpeC production and purification – HpeC was produced in RHA1 as an N-terminal polyHis-tagged (Ht-) protein using RHA1 cells containing pTip-*hpeC.* Expression was conducted as described for HpeCBA. Cells collected from 1 L of culture were suspended in 20 mL of of 50 mM sodium phosphate, 300 mM NaCl, 10 mM imidazole, pH 8, containing 2 tablets of proteinase inhibitor (cOmplete™, Mini) and DNAseI (2 μg mL^{-1}). The cell suspension was subjected to five rounds of bead-beating at 6 m/s using a FastPrep®-24 (MP Biomedicals) with 5 min on ice between rounds. Cellular debris was removed by centrifugation (40,000 × g for 40 min) and the soluble portion was filtered (0.45 μm). Ht-HpeC was purified from the cell extract using immobilized metal affinity chromatography (Ni-NTA, Thermo Fisher Scientific, Waltham, MA, USA) according to the manufacturer's protocol. The fractions containing Ht-HpeC, as judged by SDS-PAGE, were pooled and dialyzed overnight against 50 mM sodium phosphate, 150 mM NaCl, pH 8.0. Protein preparation was concentrated to ~1 mg mL^{-1}, flash frozen in liquid N_2, and stored at -80 °C until further use.

Protein analytical methods – The molecular weight and purity of the protein were analyzed using SDS-PAGE. Protein concentration was determined using the Pierce™ BCA Protein Assay Kit (Thermo Fisher Scientific, Waltham, MA, USA) with bovine serum albumin as a standard. Mass spectrometry was performed by MSL/ChiBi Proteomics Core Facility (UBC).

Streptavidin mobility shift assay – Biotinylation was determined using a previously described mobility shift assay with minor modifications[12]. Briefly, 4 μL of 10 μM HpeCBA was mixed with 2 μL of 5× PAGE loading dye and heated at 95 °C for 5 min. Samples were cooled to room temperature and 4 μL of streptavidin (5, 10, or 20 μM) were added and incubated for 5 min. Samples were then loaded onto a 15% Mini-PROTEAN SDS-PAGE gel and separated at 120 V. Running buffer was prechilled and the gel box was kept on ice during separation. Gels were stained overnight using SYPRO Ruby and imaged on a Typhoon laser scanner using a 488 nm excitation laser and a 670BP30 (655 to 685 nm) filter.

Steady-state kinetic assays – HpeHI assays were performed with 200 µM AV or HAP in 20 mM HEPPS, pH 8.0, 2 mM $MgCl_2$, 1 mM $MnCl_2$, 2 mM DTT, 1 mM ATP, and 20 µg each HpeH and HpeI at 30°C. Reactions were initiated by the addition of enzyme. Extinction coefficients for substrates and products were determined in the same buffer without ATP, as follows, ε_{340} (AV) = 12.40 mM^{-1} cm^{-1}; ε_{323} (HAP) = 11.94 mM^{-1} cm^{-1}; ε_{340} (PAV) = 0.29 mM^{-1} cm^{-1}; and ε_{323} (PAP) = 0.17 mM^{-1} cm^{-1} (SI Appendix Fig. S8). Turnover assays were performed in the same buffer with 50 µM substrate and 1 µM each HpeH and HpeI. The reaction progress was measured by change in absorbance at 323 nm for HAP and 340 nm for AV on a Cary 60 UV-Vis spectrophotometer equipped with a thermostatted cuvette holder. Rates were calculated using the $\Delta\varepsilon$ between the substrate and product. Assays were performed in triplicate.

HpeCBA activity was coupled to NADH oxidation (ε_{340} = 6.3 mM^{-1} cm^{-1}). The standard reaction was performed by incubating 266 nM HpeCBA, 0.2 mM NADH, 0.5 mM phosphoenolpyruvate, 0.5 mM ATP, 6 units of LDH and 4 units of PK (Sigma-Aldrich) in 20 mM MOPS, pH 7.5 (I = 0.1 M), 40 mM $NaHCO_3$, 4 mM $MgCl_2$, 80 mM KCl at 25 °C and was initiated by the addition of PAV or PAP. Progress curves were recorded using a Cary5000 UV-Vis spectrophotometer (Agilent Technologies). Steady-state kinetic parameters were evaluated using concentrations of substrates from 20 to 2000 µM. Data were fit to steady-state kinetic equations using LEONORA[13]. The pH optimum of the reaction was determined using 20 mM MOPS, 80 mM KCl (I = 0.1 M). HpeCBA reactions performed in the presence of HpeD were corrected for the background absorbance of end product (i.e., the phenolate anion of hydroxyphenyl-β-ketopropionate). The background absorbance was recorded by performing the reaction in the absence of NADH. These experiments were performed at pH 7.0 and 7.5 as the background absorbance was much lower at pH 7.0.

Chemical synthesis of phosphorylated compounds – The synthesis of 4-phosphoro-acetophenone (**2**) and 4-phosphoro-acetovanillone (**4**) was carried out as depicted in scheme S1, based on the synthetic route described by Silverberg et al[14]:

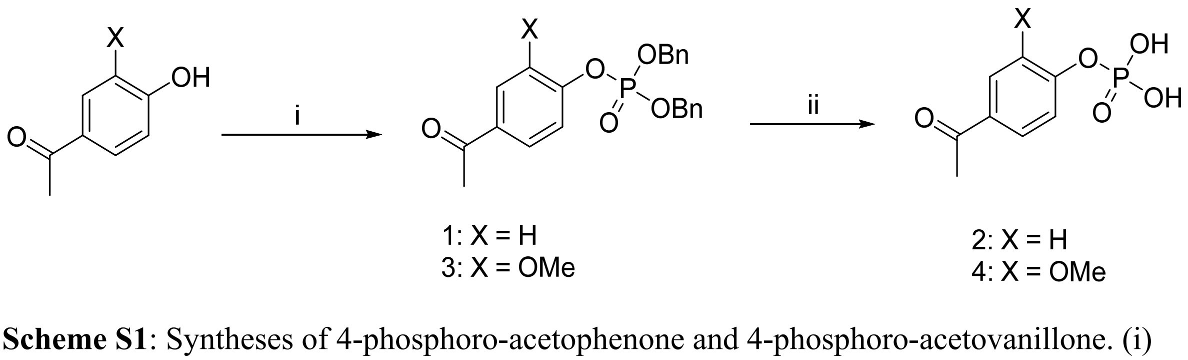

Scheme S1: Syntheses of 4-phosphoro-acetophenone and 4-phosphoro-acetovanillone. (i) CCl_4, DIPEA, DMAP, dibenzyl phosphate, Acetonitrile, -10° C. (ii) H_2, Pd/C, Ethyl acetate.

4-Hydroxyacetophenone (250 mg, 1.84 mmol) was dissolved in dry acetonitrile (9 mL) in a round bottom flask under an argon atmosphere. The solution was cooled to -10 °C and dry CCl_4 (0.88 mL, 9.2 mmol), *N,N*-diisopropylethylamine (0.67 mL, 3.86 mmol), and *N,N*-dimethylpyridin-4-amine (23 mg, 0.18 mmol) were added sequentially. Dibenzyl

phosphite (0.58 mL, 2.67 mmol) was next added to the solution dropwise and the reaction was stirred at -10 °C for 1 h until TLC indicated the consumption of the phenol. After quenching with 3 mL of 0.5 M KH_2PO_4, the mixture was extracted with ethyl acetate (15 mL × 3). The organic layers were combined and washed with water (15 mL) and brine (15 mL), dried over Na_2SO_4, filtered, and concentrated *in vacuo*. The residue was purified by silica gel column chromatography (petroleum ether: ethyl acetate = 4:1 to 1:1) to yield **1** as a clear, colorless oil (634 mg, 87%).

High Resolution-ESI-MS for $C_{22}H_{21}O_5P$: calculated 396.1127, found: 396.1130. observed ion: $C_{22}H_{21}O_5PNa^+$.

^{1}H NMR (400 MHz, $CDCl_3$) δ 7.90 (dd, J = 9.0, 0.6 Hz, 2H, Ar), 7.36 – 7.30 (m, 10H, CH_2**Ph**), 7.19 (dd, J = 8.8, 1.1 Hz, 2H, Ar), 5.13 (d, J = 8.7 Hz, 4H, **CH_2**Ph), 2.57 (s, 3H, $COCH_3$).

^{13}C NMR (101 MHz, $CDCl_3$) δ 197.2, 154.6, 135.6, 135.6 (d, J_{C-P} = 6.6 Hz), 130.8, 129.3, 129.1, 128.6, 120.5 (d, J_{C-P} = 5.2 Hz), 70.7 (d, J_{C-P} = 5.9 Hz), 27.0.

^{31}P NMR (162 MHz, $CDCl_3$) δ -6.73.

1 (480 mg, 1.13 mmol) was dissolved in 10 mL of ethyl acetate, 48 mg of Pd/C was added, and the mixture was put under an atmosphere of H_2. The reaction was stirred at room temperature for 1 hour and monitored by TLC. Upon completion, the reaction mixture was filtered over Celite and concentrated *in vacuo*. The mixture was then purified by reverse phase chromatography (Waters C18 Sep-Pak, 20 cc Vac Cartridge, 5 g Sorbent per Cartridge, 37-55 μm) using water and acetonitrile as eluent to yield **2** as a light brown solid (178 mg, 0.82 mmol, 73%). This compound was found to contain a small amount of an impurity with very similar retention time to the product. Low resolution ESI-MS suggests that it is the compound resulting from reduction of the ketone to alcohol.

High Resolution-ESI-MS for $C_8H_9O_5P$: calculated 216.0888, found: 216.0888. observed ion: $C_8H_{10}O_5P^+$.

^{1}H NMR (300 MHz, D_2O) δ 7.90 (d, J = 8.8 Hz, 2H, Ar), 7.21 (d, J = 8.5 Hz, 2H, Ar), 2.53 (s, 3H, $COCH_3$).

^{13}C NMR (75 MHz, D_2O) δ 202.9, 156.4 (d, J_{C-P} = 6.6 Hz), 132.5, 130.9, 120.3 (d, J_{C-P} = 4.9 Hz), 26.2.

^{31}P NMR (122 MHz, D_2O) δ -5.33.

Starting from acetovanillone (150 mg, 0.90 mmol) and following the same procedure as for synthesis of compound **1**, **3** was synthesized (280 mg, 0.66 mmol, 73%).

High Resolution-ESI-MS for $C_{23}H_{23}O_6P$: calculated 426.1233, found: 426.1232. observed ion: $C_{23}H_{23}O_6PNa^+$.

^{1}**H NMR** (400 MHz, $CDCl_3$) δ 7.55 (dd, *J* = 1.9, 1.4 Hz, 1H, Ar), 7.47 (dd, *J* = 8.3, 2.0 Hz, 1H, Ar), 7.37 – 7.32 (m, 10H, CH_2**Ph**), 7.30 (dd, *J* = 8.3, 1.4 Hz, 1H, Ar), 5.19 (d, *J* = 8.2 Hz, 4H, $\mathbf{CH_2}$Ph), 3.85 (s, 3H, OCH_3), 2.58 (s, 3H, $COCH_3$).

^{13}**C NMR** (101 MHz, $CDCl_3$) δ 196.8, 150.8 (d, $J_{C\text{-}P}$ = 5.5 Hz), 143.6 (d, $J_{C\text{-}P}$ = 7.0 Hz), 135.4 (d, $J_{C\text{-}P}$ = 7.1 Hz), 134.8 (d, $J_{C\text{-}P}$ = 1.4 Hz), 128.6, 128.6, 128.0, 122.1 (d, $J_{C\text{-}P}$ = 1.7 Hz), 121.0 (d, $J_{C\text{-}P}$ = 3.0 Hz), 111.6, 70.1 (d, $J_{C\text{-}P}$ = 5.9 Hz), 56.0, 26.5.

^{31}**P NMR** (162 MHz, $CDCl_3$) δ -6.48.

Compound **3** (50 mg, 0.12 mmol) was subjected to the same deprotection procedure detailed above to yield **4** as the product in sufficient purity (18 mg, 0.076 mmol, 65% yield, ~95% pure). Similar to the case for compound **1**, an impurity resulting from reduction of the ketone functional group was observed, but the amount of this impurity was more noticeable in this case, about 5% as estimated by H-NMR. The product was therefore further purified by HPLC to yield pure compound for analytical purposes. (Column: Agilent Eclipse XBD-C18, 5 µm, 46 mm ID x 250 mm, Mobile phase: Acetonitrile (0.1% TFA) /Water (0.1% TFA) 5:95 to 20:80, 13 min, Flow rate: 3 mL/min).

High Resolution-ESI-MS for $C_9H_{11}O_6P$: calculated 246.0293, found: 246.0292. observed ion: $C_9H_{12}O_6P^+$.

^{1}**H NMR** (600 MHz, D_2O) δ 7.64 (dd, *J* = 8.4, 2.0 Hz, 1H, Ar), 7.57 (d, *J* = 2.0 Hz, 1H, Ar), 7.41 (d, *J* = 8.4 Hz, 1H, Ar), 3.90 (s, 3H, OCH_3), 2.62 (s, 3H, $COCH_3$).

^{13}**C NMR** (151 MHz, D_2O) δ 201.9, δ 149.6 (d, $J_{C\text{-}P}$ = 5.5 Hz), 145.3 (d, $J_{C\text{-}P}$ = 6.4 Hz), 132.2, 122.7, 119.8 (d, $J_{C\text{-}P}$ = 1.6 Hz), 111.6, 55.4, 25.4.

^{31}**P NMR** (162 MHz, D_2O) δ -3.05.

LC/MS Analysis – Reactions of HpeCBA with PAV or PAP, as described above were quenched by addition of an equivalent volume of methanol. Reactions of HpeHI with HAP or AV, were performed in 20 mM HEPPS, pH 8.5, 2 mM $MgCl_2$, 1 mM $MnCl_2$, 2 mM DTT, 1 mM ATP, 200 µg of HpeH, 200 µg HpeI and 200 µM HAP or AV. Samples were incubated at 30°C for 5 min and, were quenched with the addition of 10% acetic acid. LC-MS analysis performed using an Agilent 1290 Infinity II UHPLC in line with an Agilent 6546 Q-TOF with a dual AJS ESI source. Five µL samples were injected onto a Zorbax Eclipse Plus c18 column (100 mm × 2.1 mm × 1.8 µM) and run on a 20 min linear gradient from 5 to 100% solvent B at 0.45 mL min^{-1}. Solvent A was 0.1% formic acid in water, solvent B was 0.1% formic acid in methanol. MS parameters in negative ionization mode were as follows: capillary voltage, 4000 V; nozzle voltage, 2000 V; drying gas temp, 300 °C; drying gas flow rate, 10 L min^{-1}; sheath gas temperature, 350 °C; sheath gas flow rate 12 L min^{-1}, nebulizer pressure, 45 psi; fragmentor voltage, 100 V. Parameters for positive ionization mode were the same, except capillary voltage, 3500 V and nozzle voltage, 500 V. MS/MS was collected on selected ions with 10, 20, and 40

V collision energies. Data were collected and analyzed using MassHunter Workstation Version 10.

Characterization of OCF extracts – The wood sample was ground into a powder with a knife, milled so it could pass through a 40-mesh screen, and subsequently Soxhlet extracted with acetone to make extractive free wood powder. 10 g of dried wood powder was dispersed in 500 mL of 7.5% NaOH solution, placed in stirred Parr reactor and pressurized with O_2 to 1 MPa after purging the reactor. The samples were stirred at 400 RPM for 1 h at 160 °C. The reaction slurry was acidified with HCl to pH 2 and then further extracted. Compounds were extracted from the OCF products using ethyl acetate, in 25:10 ratio (OCF:solvent, v/v) and dehydrated with Na_2SO_4 prior to analysis. Note, no solid residuals were detected after the OCF treatment.

Aromatic compounds in extracts were identified using an Agilent Technologies (Santa Clara, U.S.A.) 6890N gas chromatograph equipped with a 30-m Agilent 190915-433 capillary column and an Agilent 5973 mass-selective detector. Samples were dried and derivatized using *N*,*O*-bis(trimethylsilyl)trifluoroacetamide and trimethylchlorosilane in a 50/50 mixture with pyridine. Runs were held at 90 °C for 3 min, and then ramped to 290 °C at 12 °C min^{-1} with a 10 min final hold. Authentic standards of vanillin, acetovanillone, vanillate, lactate, gylocate and fumarate were run in parallel. Aliphatic acids were quantified by GC-MS using standard curves. For monoaromatics quantification in OCF slurry and extracts, HPLC analysis was performed using a Waters 2695 HPLC (Waters, Milford, MA) equipped with a 250 × 4.6 mm Luna® 5 µm C18 column (Phenomenex, Torrance, CA) and a UV detector, as described above. OCF slurry samples were diluted 1:100 in 10% acetic acid and centrifuged for 5 min at maximum velocity. Filtered (0.2 µm) samples were injected. For OCF extracts, ethyl acetate was evaporated, the extracts suspended in methanol, filtered (0.2 µm) and injected. Vanillin, acetovanillone and vanillate were quantified by HPLC using standard curves.

GD02 growth on OCF extracts – For growth of GD02 on OCF extracts, a single colony was inoculated in 5 mL LB broth and grown overnight. Cells were pelleted at 1000 RCF, washed twice with M9, then used to inoculate M9+minerals at OD_{600} ~0.05. For toxicity experiments, LB broth was amended with different amounts of OCF extracts and cells inoculated at OD_{600} ~0.05. LB without OCF extract served as a control. For substrate preparation, solvent was evaporated under N_2 and suspended in DMSO to prepare a stock solution. Growth studies in 96-well plate were performed using a Tecan Spark-Multimode Microplate Reader with shaking at 250 rpm and OD_{600} recorded every 30 min. For growth experiments in flasks, cells were incubated with 2 mM (monoaromatic compounds) OCF extracts at 200 rpm, and the growth was followed by measuring OD_{600}. To evaluate the monoaromatic compounds depletion in culture supernatant, 100-µL samples were withdrawn, acidified to 10% acetic acid, and processed and analyzed by HPLC as described above. To evaluate the small acids depletion, 300-µL samples were withdrawn, acidified with 10% acetic acid, extracted with equal volume of ethyl acetate, and processed and analyzed by GC-MS as described above

SI Figures

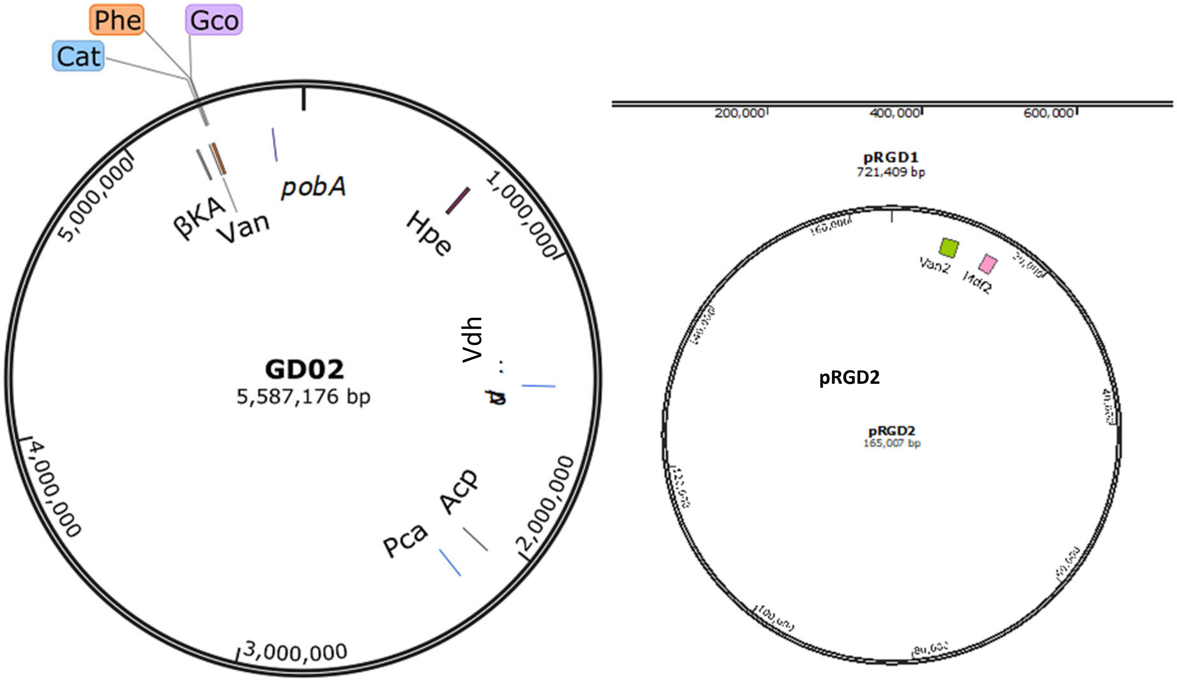

Figure S1. Genomic elements of *Rhodococcus rhodochrous* GD02. Most of the aromatic compound catabolism genes (Table 1, Table S1) are concentrated in one region of the chromosome, but the *hpe, acp, vdh* and *pcaGH* genes are not in this region. Gene cluster abbreviations: Pca, protocatechuate; MDF, methionine-dependent formaldehyde oxidation, βKA, beta-ketoadipate; Van, vanillate; Cat, catechol; Phe, phenol; Gco, guaiacol.

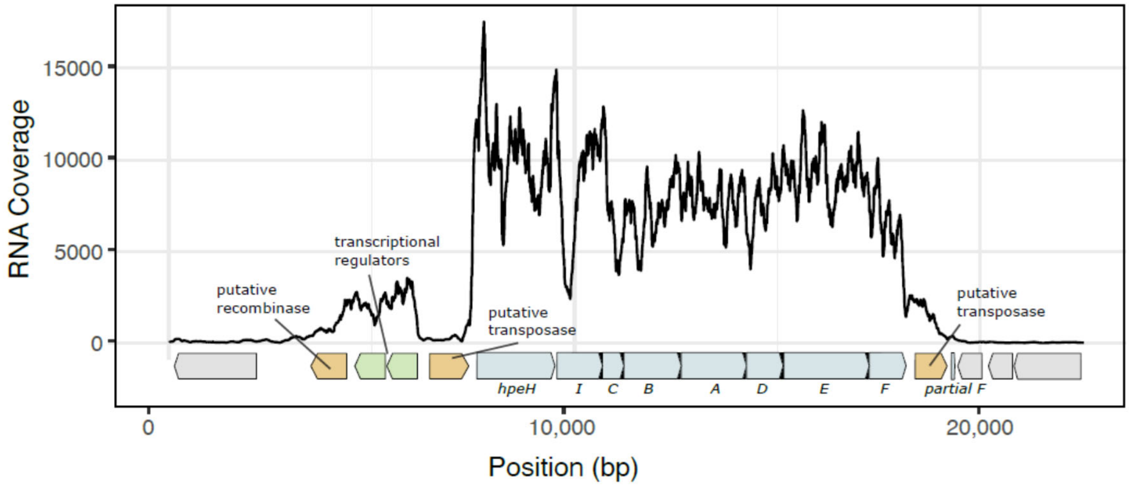

Figure S2. Evidence for co-transcription and horizontal transfer of the *hpe* gene cluster. The graph shows transcriptional read coverage in the region of the *hpe* gene cluster during growth of GD02 on AV. Coverage of junctions between *hpe* genes indicates co-transcription of the entire region, but relatively low coverage of the 5-prime end of *hpeI* suggests factors may affect transcriptional regulation of individual genes. Dark shading at the junctions of *hpeICBADEF* indicates four-nucleotide overlaps of the putative open reading frames at each of these junctions. These overlaps provide additional evidence for co-transcription of these genes. The putative transcriptional regulators encoded upstream of the *hpe* gene cluster are IclR family transcriptional regulators that may modulate transcription of *hpe* genes. Transposase and recombinase genes flanking the *hpe* gene cluster suggest horizontal transfer of the cluster. The GC content of the GD02 chromosome and the *hpe* gene cluster are 68% and 67%, respectively, which is probably too small a difference to be evidence for horizonal transfer.

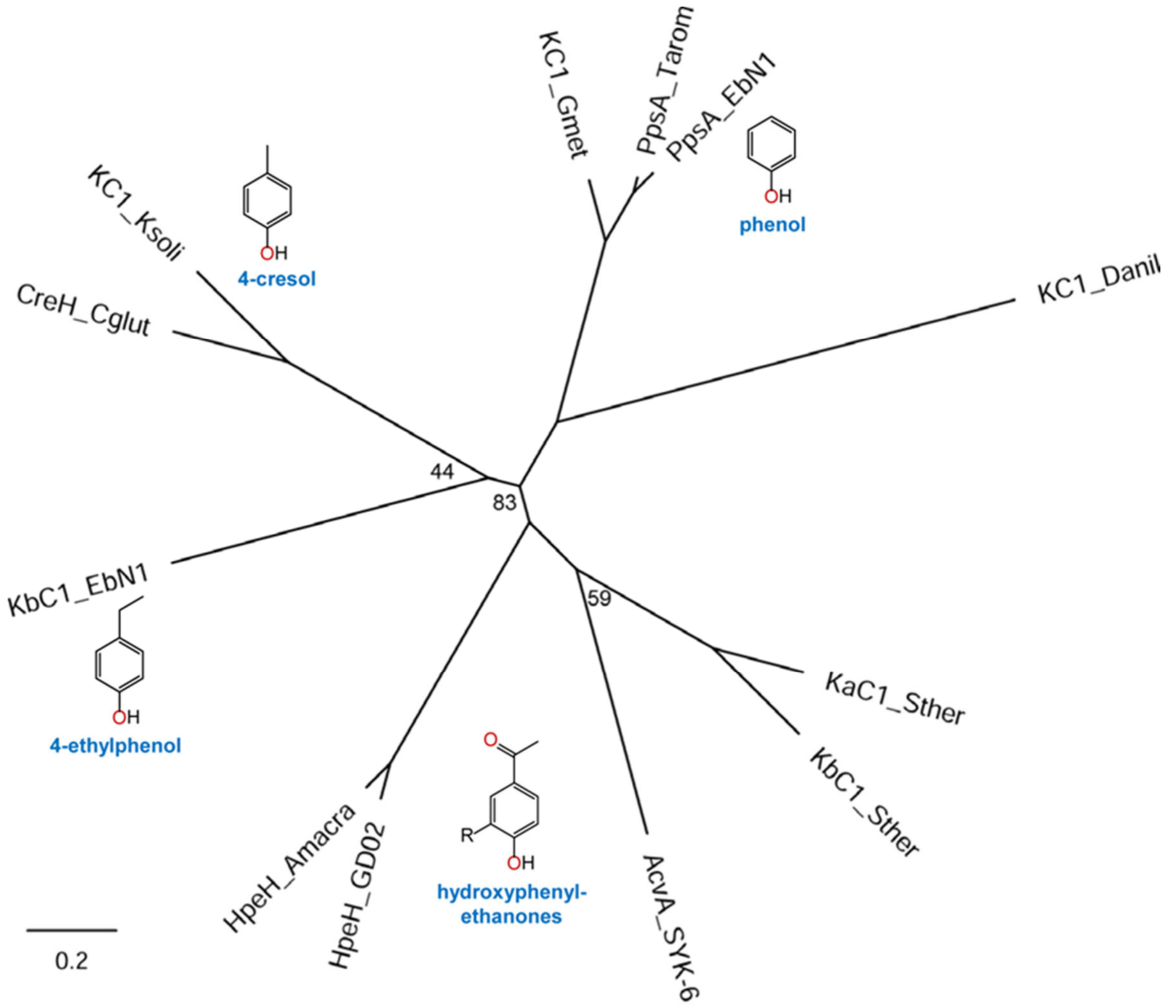

Figure S3. Phylogeny of HpeH homologs. Homlogs are: HpeH_GD02 (WP_229583467), *Rhodococcus rhodochrous* GD02; HpeH_Amacra (WP_067449674), *Actinomadura macra* NBRC 14102; KC1_Ksoli (WP_091241005*), Klenkia soli* DSM 45843, CreH_Cglut (WP_011013724), *Corynebacterium glutamicum* ATCC 13032; KC1_Danil (WP_051184705), *Desulfatiglans anilini* DSM 4660; PpsA_Tarom (WP_107221732), *Thauera aromatica* AR-1; KbC1_EbN1 (WP_041646519), *Aromatoleum aromaticum* EbN1; PpsA_EbN1 (WP_011237602), *A. aromaticum* EbN1; KC1_Gmet (WP_004513104), *Geobacter metallireducens* GS-15; AvcA_SYK-6 (WP_014074980), *Sphingobium* sp. SYK-6; KaC1_Sther (WP_012873266), *Sphaerobacter thermophilus* DSM 20745; KbC1_Sther (WP_012873268), *Sphaerobacter thermophilus* DSM 20745. KC denotes kinase components whose substrates are unknown. Where the substrate is known, the structure is indicated adjacent to the relevant homolog(s). Bootstrap values greater than 90 are not indicated.

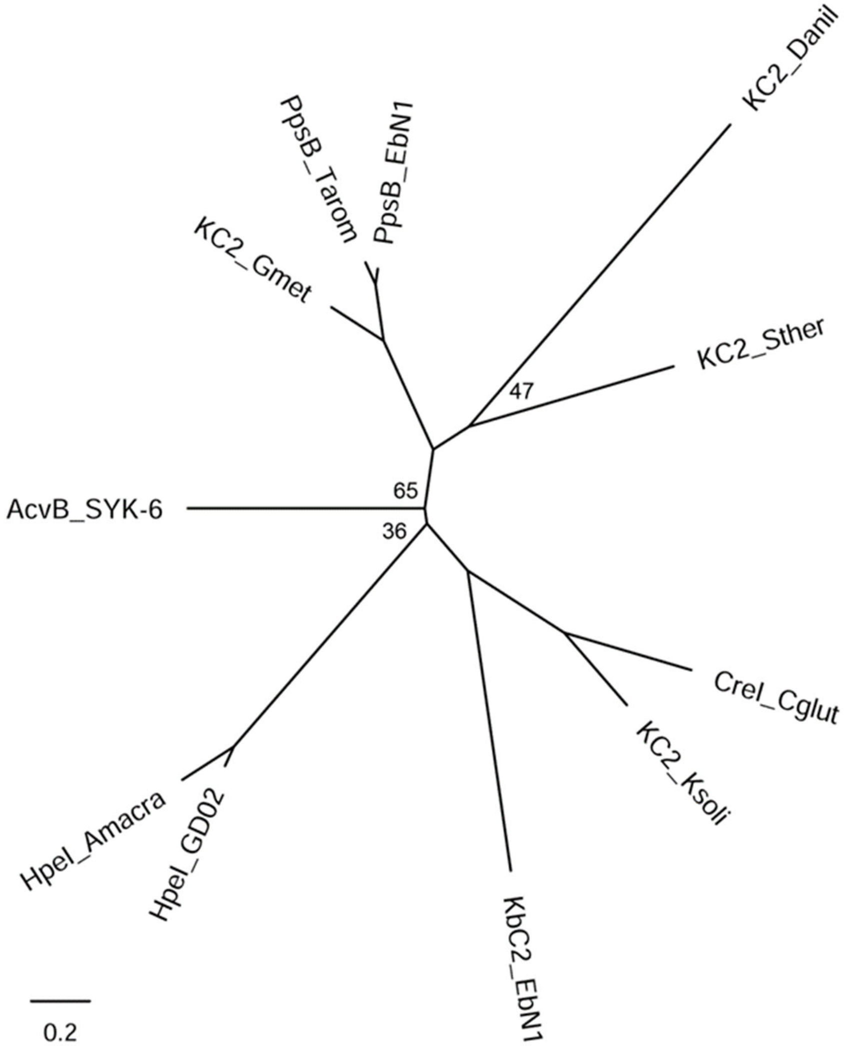

Figure S4. Phylogeny of HpeI homologs. Homlogs are: HpeI_GD02 (WP_229581529), *R. rhodochrous* GD02; HpeI_Amacra (WP_067449672), *A. macra* NBRC 14102; KC2_Ksoli (WP_207500316), *K. soli* DSM 45843; CreI_Cglut (WP_011013725), *C. glutamicum* ATCC 13032; KC2_Danil (WP_161626600), *D. anilini* DSM 4660; PpsB_Tarom (WP_107221733), *T. aromatica* AR-1; KbC2_EbN1 (WP_011239072), *A. aromaticum* EbN1; PpsB_EbN1 (WP_011237601), *A. aromaticum* EbN1; KC2_Gmet (WP_004513103), *G. metallireducens* GS-15; AvcB_SYK-6 (WP_014074979), *Sphingobium* sp. SYK-6; KC2_Sther (WP_012873267), *S. thermophilus* DSM 20745. KC denotes kinase components whose substrates are unknown. Bootstrap values greater than 90 are not indicated.

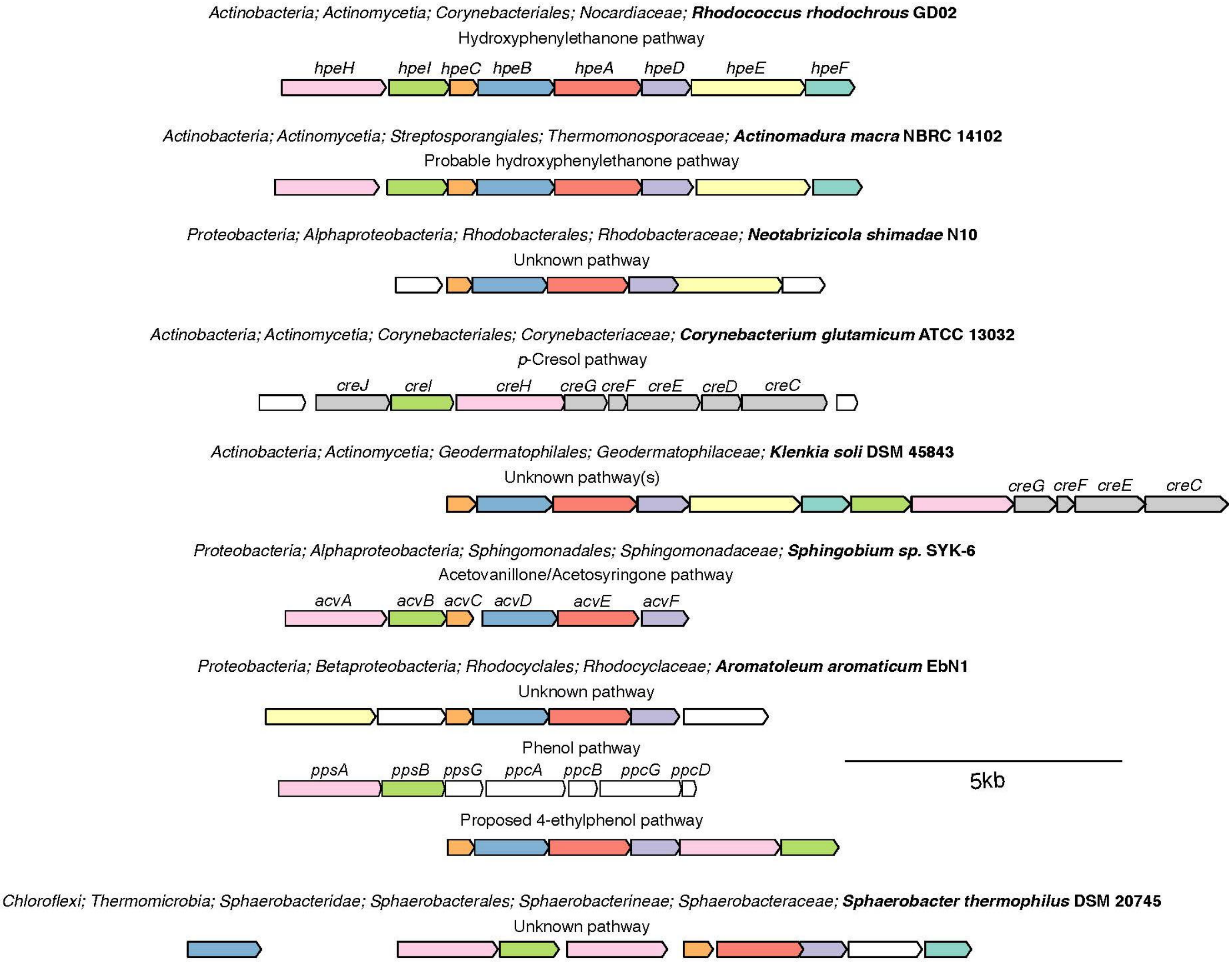

Figure S5. Representative gene clusters from diverse bacteria containing various homologs of *hpe* genes. Color coding indicates homology with specific *hpe* genes. The uncolored genes in the EbN1 phenol pathway cluster encode PpsG, a third component of phenylphosphate synthase that does not have a homolog in hydroxyphenylethanone kinase, and PccABGD, phenol carboxylase, which is not homologous to phosphophenylethanone carboxylase. Grey genes in the DSM 45843 cluster are homologous with *cre* genes in ATCC 13032.

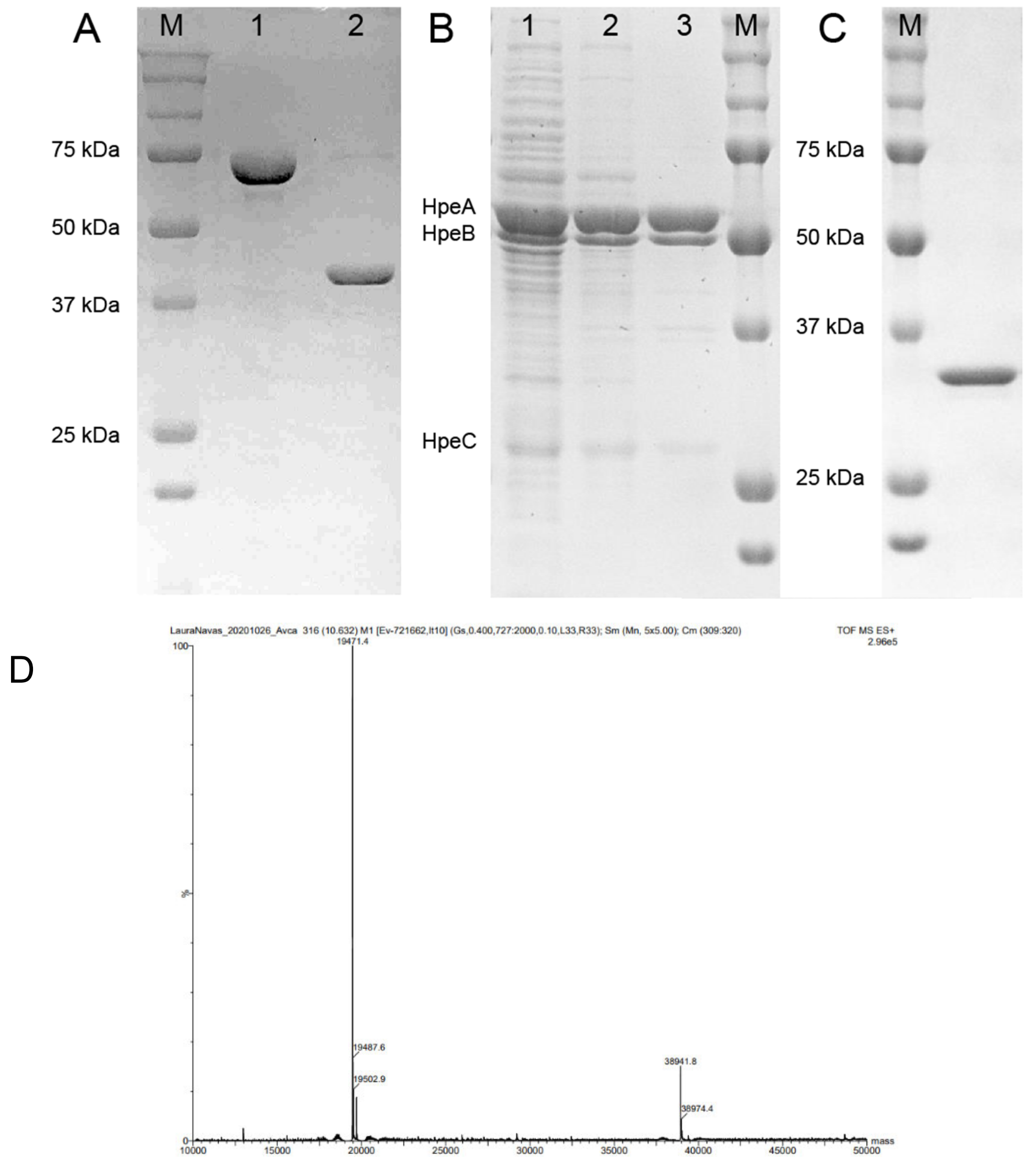

Figure S6. SDS-PAGE analyses of HpeHI, HpeCBA and HpeD preparations. (**A**) Purified His tagged (Ht-)HpeH and Ht-HpeI from *E. coli* BL21 λ (DE3) harboring pET-*hpeH* and pET-*hpeI* using immobilized metal affinity chromatography. 1: eluate with HpeH, 2: eluate with HpeI. (**B**) Purified HpeCBA complex from RHA1 harboring pTip-*hpeCBA*. 1: lysate of RHA1 harboring pTip-*hpeCBA*, 2: proteins precipitated from the lysate using 1.3 M ammonium sulfate, 3: HpeCBA complex purified after anion exchange chromatography, M: Molecular Weight Marker. (**C**) Purified Ht-HpeD from *E. coli* BL21 λ (DE3) harboring pET-*hpeD* using immobilized metal affinity chromatography. 1: eluate with HpeD. (**D**) Mass spectrum of Ht-HpeC.

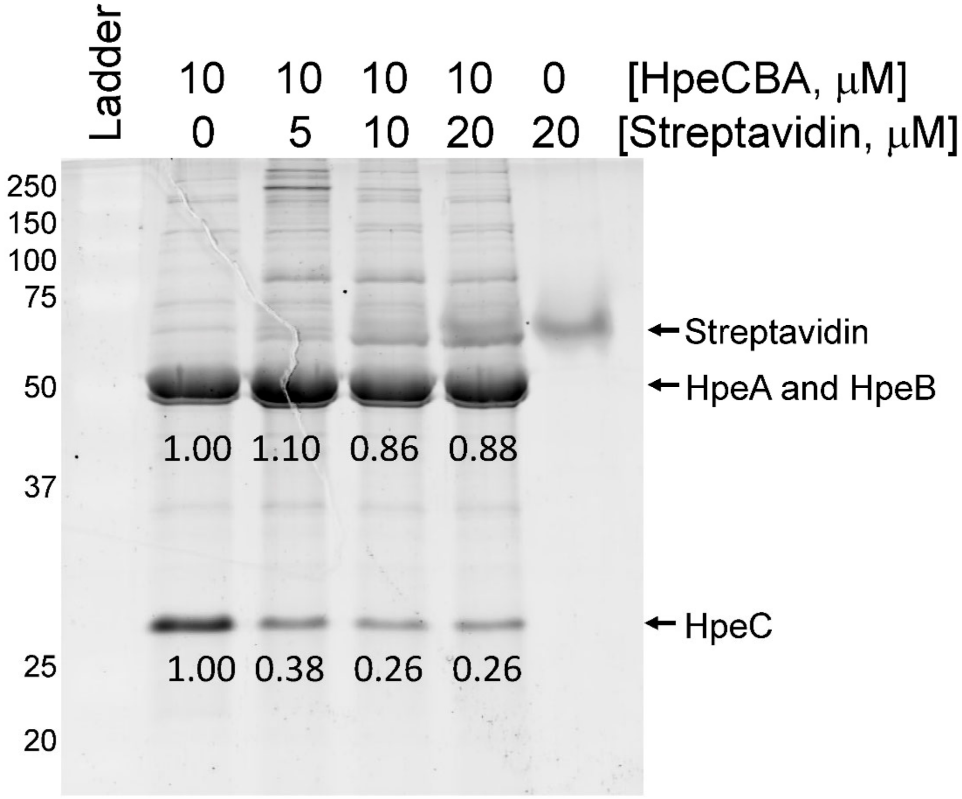

Figure S7. Streptavidin-shift assay of HpeCBA. Purified HpeCBA was denatured and incubated with 0.5 to 2 molar equivalents of Streptavidin prior to separation by SDS-PAGE. The gel was stained with SYPRO Ruby and imaged with excitation at 488 nm with a 655-685 nm emission filter to quantitate band intensity. The pre-stained PAGE ladder did not fluoresce in this range, so markers are indicated by molecular weights on the left. HpeBA were poorly resolved under these running conditions, so were quantitated as one species. Intensity for HpeBA and HpeC are indicated relative to the intensity in sample lane 1 containing HpeCBA alone.

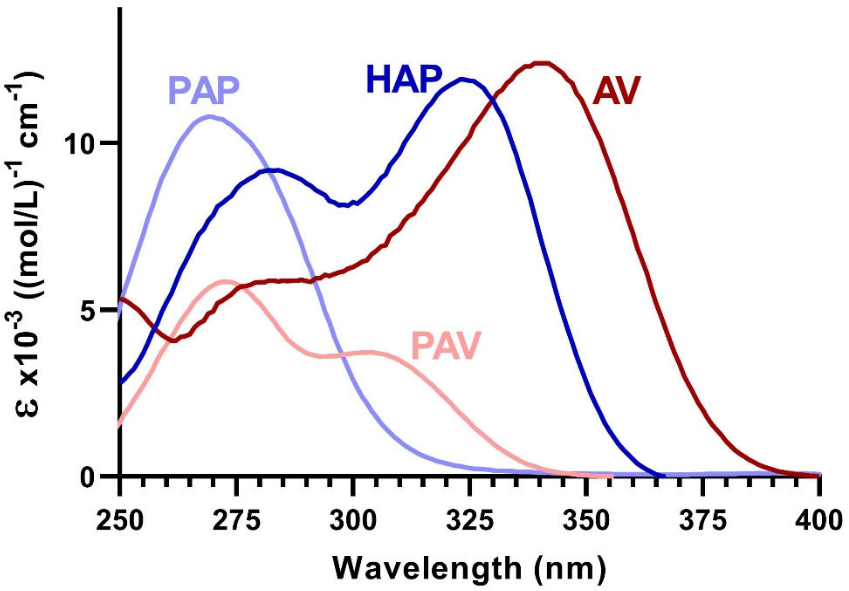

Figure S8. UV-visible spectra of HAP, AV, PAP and PAV. Compounds were in 20 mM HEPPS, pH 8.0, 2 mM $MgCl_2$, 1 mM $MnCl_2$, 2 mM DTT.

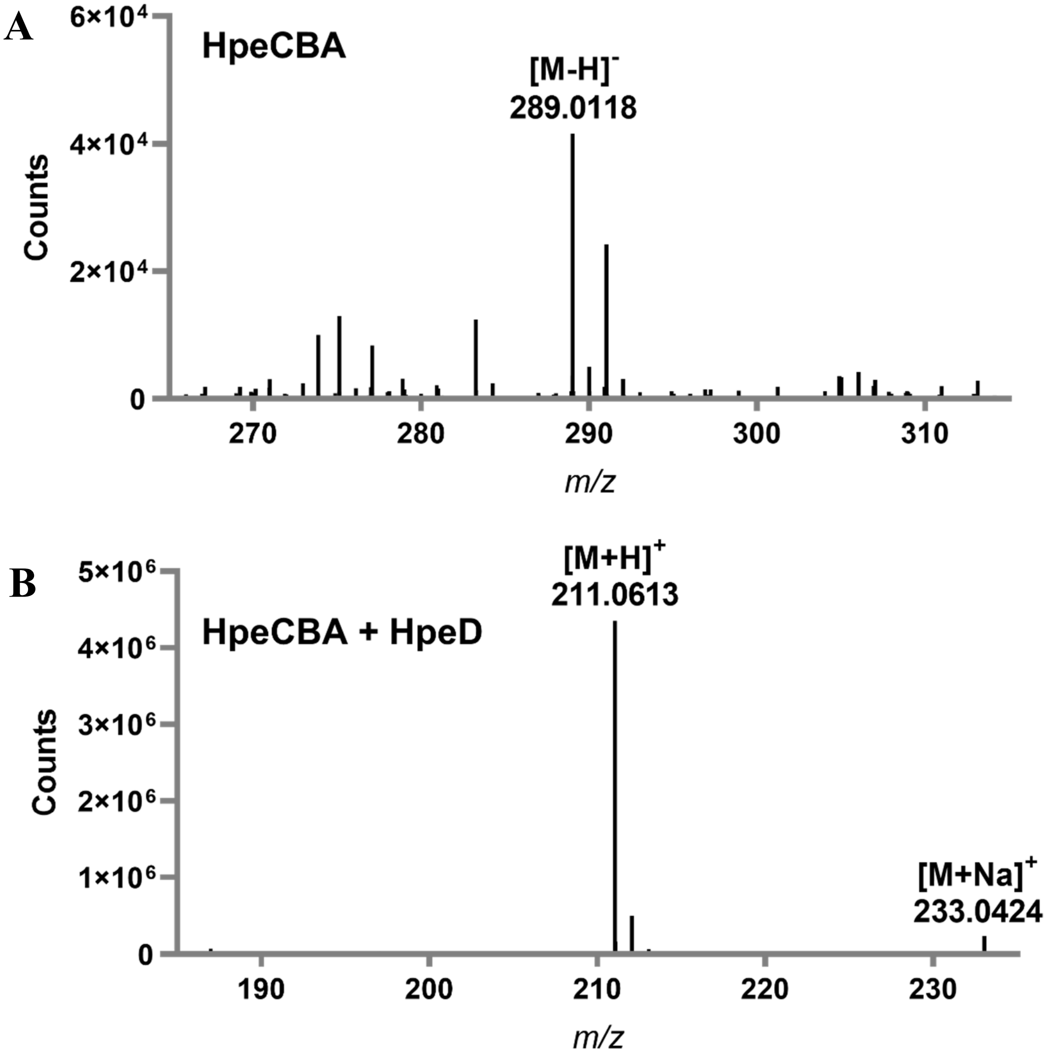

Figure S9. LC-MS Mass spectra of products of reactions catalyzed by HpeCBA and HpeD using 4-phosphoacetovanillone (PAV) as substrate. Observed *m/z* values match theoretical values for (**A**) 4-phosphocarboxyacetovanillone (PCAV) and (**B**) 4-hydroxy-3-methoxyphenyl-β-ketopropionate. Values are within 6 ppm error.

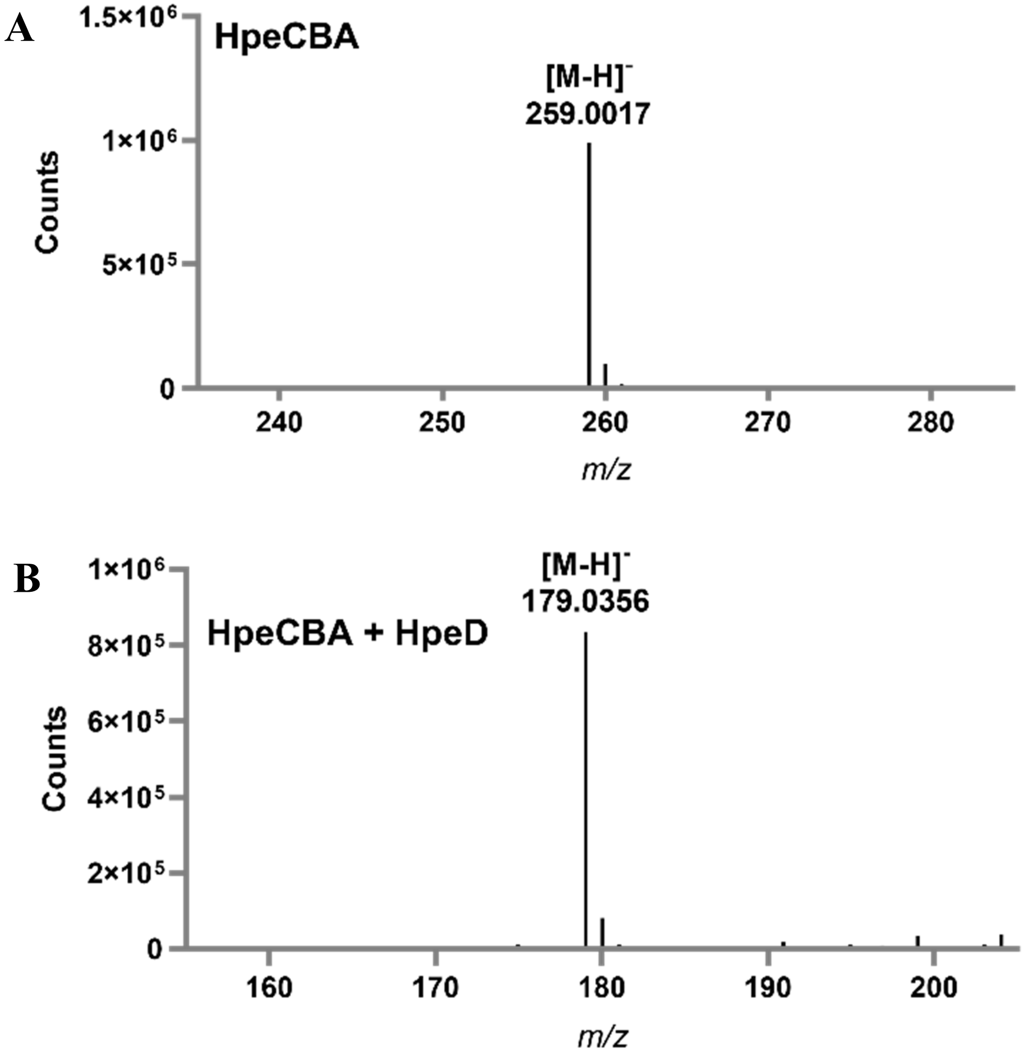

Figure S10. LC-MS Mass spectra of products of reactions catalyzed by HpeCBA and HpeD using 4-phosphoacetophenone (PAP) as substrate. Observed *m/z* values match theoretical values for (**A**) 4-phosphocarboxyacetophenone (PCAP) and (**B**) 4-hydroxyphenyl-β-ketopropionate. Values are within 4 ppm error.

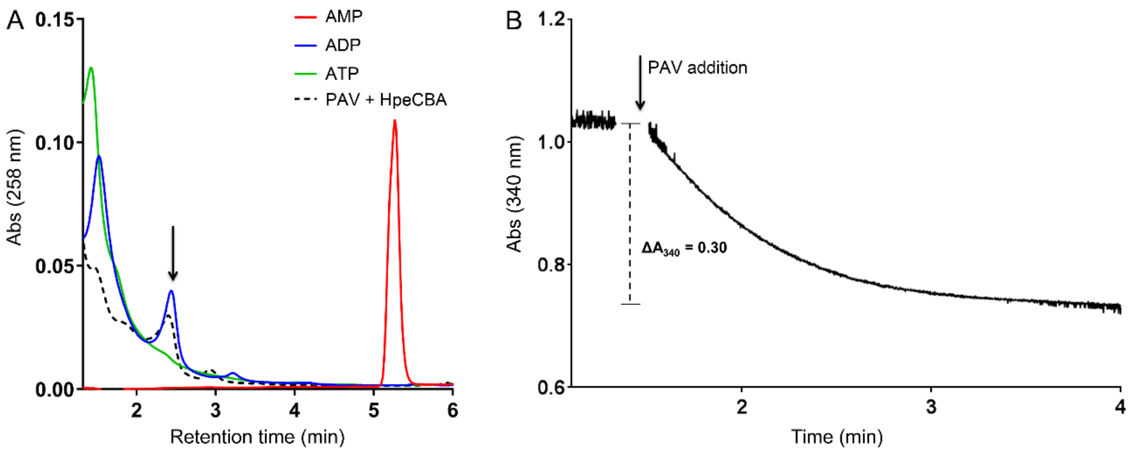

Figure S11. ATP hydrolysis to ADP by HpeCBA. (**A**) HPLC traces of standards of ATP, ADP, and AMP are shown in colors. The reaction product of PAV treated with HpeCBA in presence of ATP, HCO_3^- and Mg^{+2} is shown in dotted line. Arrow indicates ADP generation. (**B**) Stoichiometry of ATP hydrolysis. A coupled enzyme was started with a limiting amount of PAV (50 µM). The absorbance difference (ΔA_{340}) between the start of the reaction and its leveling off is represented in dotted line and was used to calculate the amount of oxidized NADH. Solid arrow indicates the time point when the substrate was added.

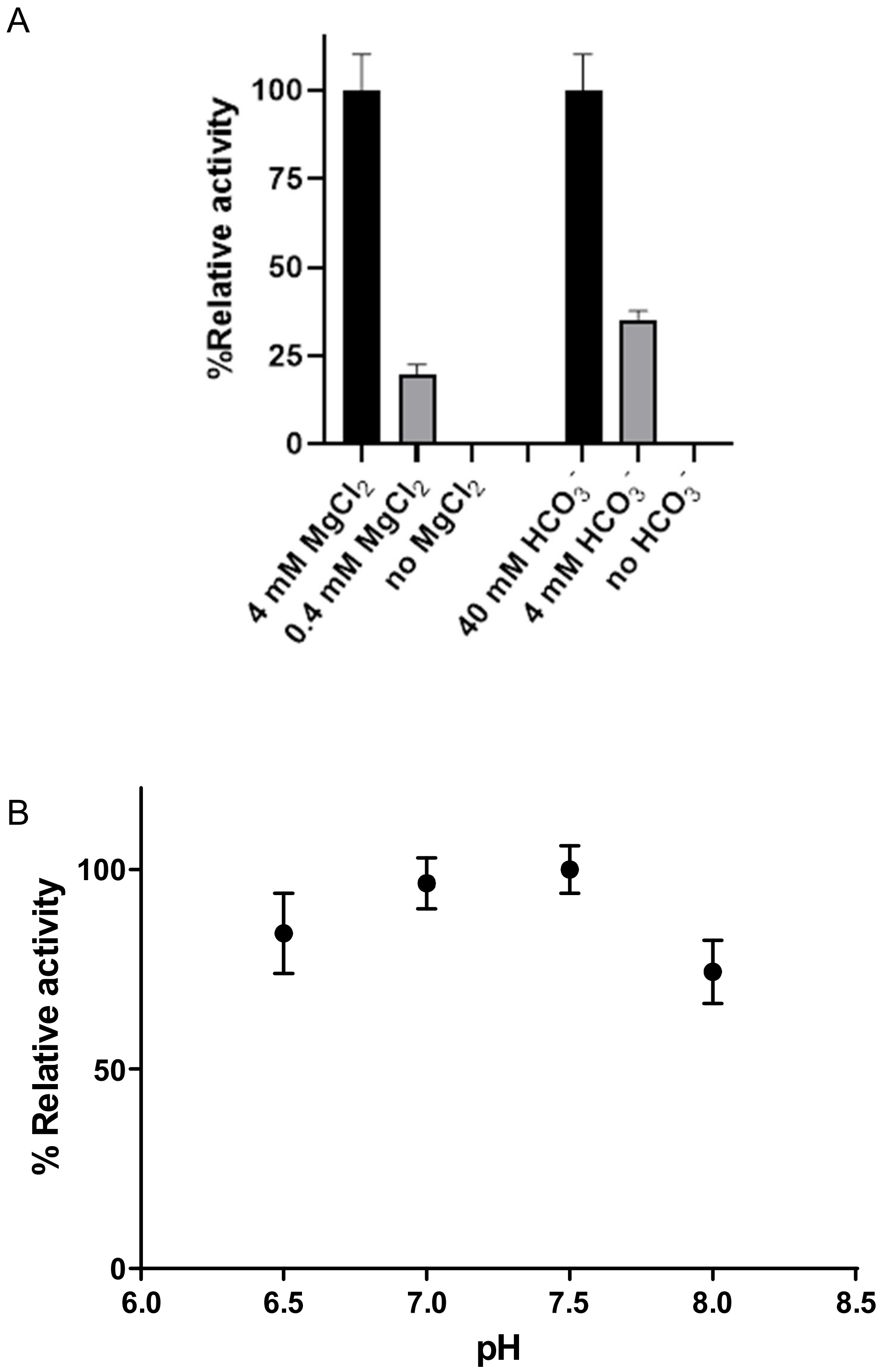

Figure S12. Characterization of HpeCBA activity in coupled reactions. Reactions were performed under standard conditions unless otherwise stated. (**A**) Dependence of activity on $MgCl_2$ and bicarbonate. Error bars indicate standard error for duplicates. (**B**) Dependence of activity on pH. The buffer was 20 mM MOPS, 80 mM NaCl (I = 0.1 M). Error bars indicate standard deviation of triplicates.

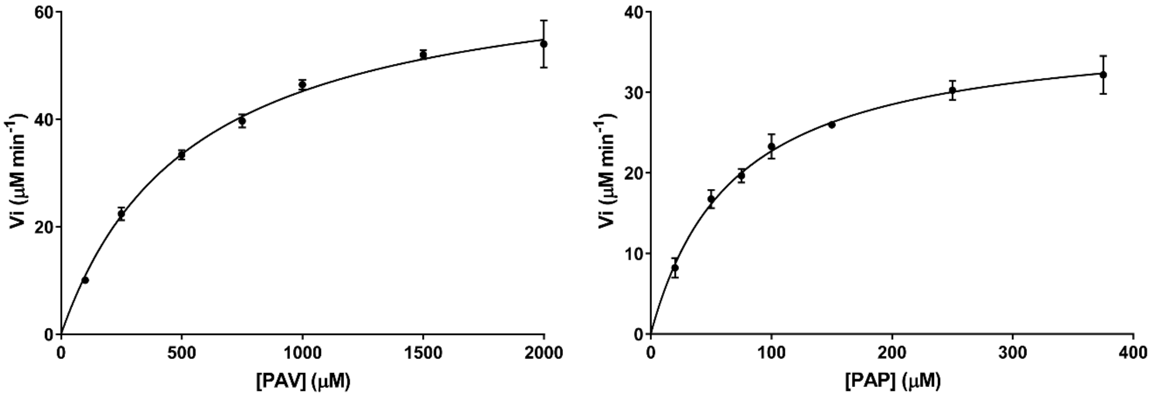

Figure S13. Steady-state kinetic analyses of the HpeCBA-catalyzed reactions. Dependence of initial velocity on substrate concentration in 20 mM MOPS (I = 0.1 M), pH 7.5, 25 °C. Data points represent the average of triplicate experiments, and the error bars the standard deviation. Curves represent fit of the Michaelis-Menten equation to the data. PAV, 4-phosphoacetovanillone; PAP, 4-phosphoacetophenone.

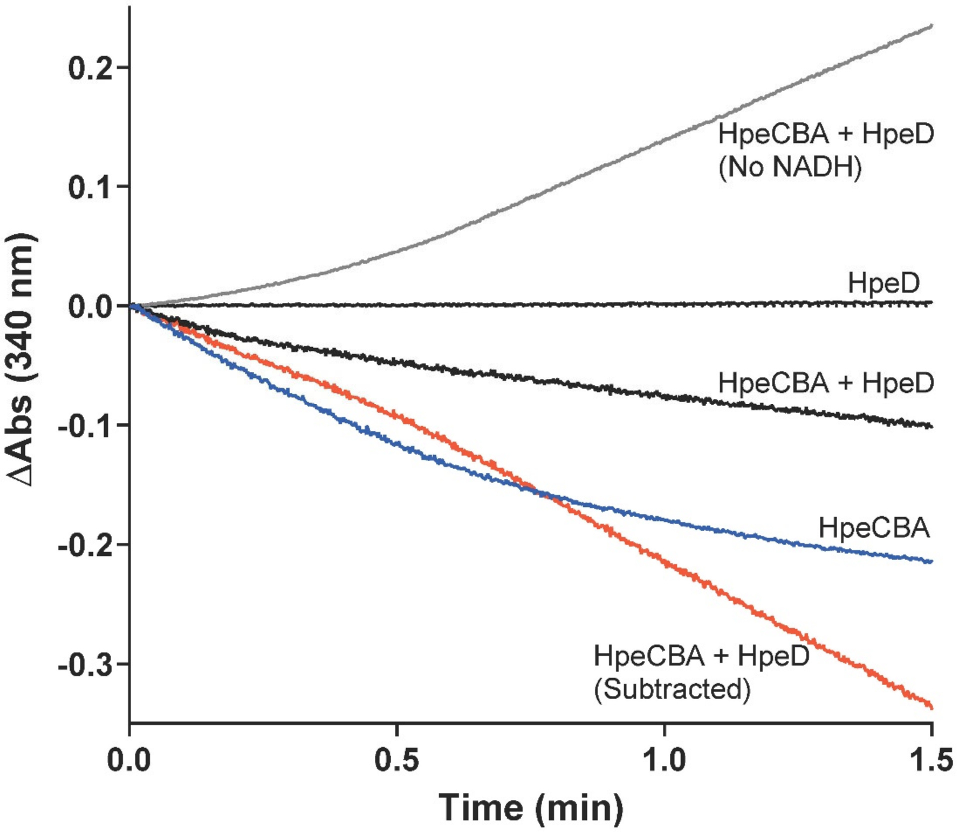

Figure S14. The effect of HpeD on the turnover of HpeCBA. Reactions were performed and monitored as described for the coupled HpeCBA unless otherwise indicated. The reaction buffer was 20 mM MOPS (I = 0.1 M), pH 7.0. Reactions contained HpeCBA (0.25 μM) and/or HpeD (0.25 μM) as indicated, and were initiated by the addition of PAP to 345 μM. Reactions performed in the absence of HpeD (blue trace) showed faster rates of A_{340} decrease than in its presence (black trace). However, a control reaction containing no NADH (grey trace) showed a strong increase in A_{340} due to the absorbance of HAPC, the dephosphorylated product of the HpeD-catalyzed reaction, whose production was confirmed by HPLC (Fig. 4). Correcting the black trace for this absorbance yielded the red trace. Maximal rates calculated from the blue and red traces were similar (see text). Reactions were performed in triplicate to calculate the specific activity. Single representative traces are shown. Similar results were obtained at pH 7.5. However, HAPC absorbs more strongly at pH 7.5, so reactions were performed at pH 7.0 to mitigate this absorbance HAPC.

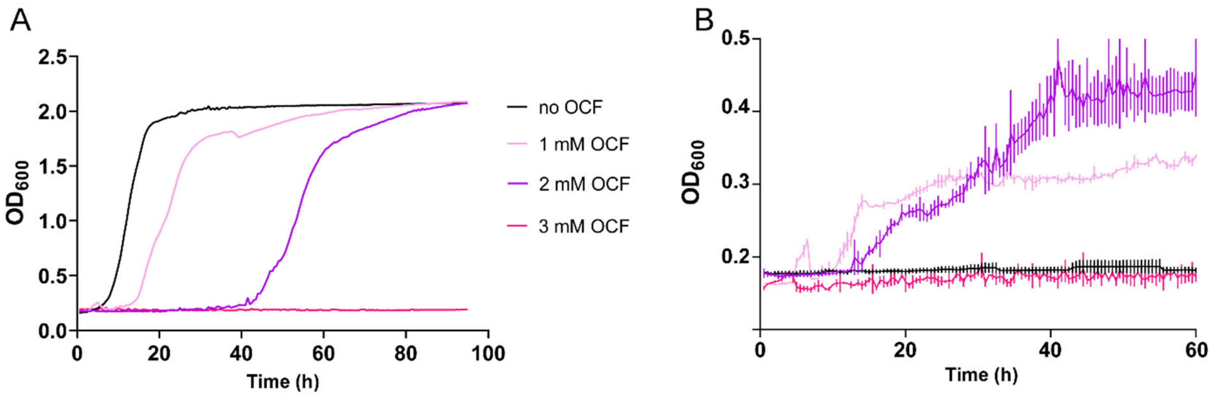

Figure S15. Inhibition and growth of GD02 with OCF extracts. (**A**) Inhibition of growth on LB by adding different concentrations (referring to total monoaromatic compounds) of OCF extracts. Curves represent single replicates that are representative of triplicate experiments. (**B**) Growth on different concentrations of OCF extracts in defined medium. OD_{600} was monitored continuously at 30 °C using a plate reader. Solid lines represent the average of three replicates. The vertical bars indicate the standard deviation.

SI Tables

Table S1. Genes encoding aromatic compound degradation identified in GD02[a]

Gene cluster[b]	Gene name	GD02 Locus	Annotation	Ortholog[c]	%ID[d]
	vdh	LCH94_06640	vanillin dehydrogenase	RHA1_ro02986	52
Pca	*pcaG*	LCH94_10080	protocatechuate 3,4-dioxygenase, α subunit	RHA1_ro01336	41
	pcaH	LCH94_10085	protocatechuate 3,4-dioxygenase, β subunit	RHA1_ro01335	49
Mdf	*fmhA*	LCH94_16935	*S*-formyl-mycothiol hydrolase	RHA1_ro02586	76
	adhE	LCH94_16940	mycothiol-dependent formaldehyde dehydrogenase	RHA1_ro02587	88
βKA	*pcaJ*	LCH94_23695	β-ketoadipate CoA-transferase, β subunit	RHA1_ro01333	77
	pcaI	LCH94_23700	β-ketoadipate CoA-transferase, α subunit	RHA1_ro01334	81
	pcaB	LCH94_23705	3-carboxy-cis,cis-muconate cycloisomerase	RHA1_ro01337	59
	pcaL	LCH94_23710	β-ketoadipate enol-lactonase	RHA1_ro01338	63
	pcaR	LCH94_23715	regulatory protein	RHA1_ro01339	71
	pcaF	LCH94_23720	β-ketoadipate:succinyl-CoA thiolase	RHA1_ro01340	81
Van	*vanA*	LCH94_23920	vanillate *O*-demethylase, oxygenase	RHA1_ro04165	35
	vanB	LCH94_23925	vanillate *O*-demethylase, reductase	RHA1_ro04163	39
	vanK	LCH94_23930	MFS Transporter	RHA1_ro02923	50
Cat	*catC*	LCH94_23960	muconolactone *δ*-isomerase	RHA1_ro02371	90
	catB	LCH94_23965	muconate cycloisomerase	RHA1_ro02372	76
	catA	LCH94_23970	catechol 1,2-dioxygenase	RHA1_ro02373	69
Phe	*pheB*	LCH94_23985	phenol 2-monooxygenase, reductase	RHA1_ro08076	75
	pheA	LCH94_23990	phenol 2-monooxygenase, oxygenase	RHA1_ro08077	83
Gco	*gcoA*	LCH94_24005	guaiacol *O*-demethylase, oxygenase	RHA1_ro02382	76
	gcoB	LCH94_24010	guaiacol *O*-demethylase, reductase	RHA1_ro02383	60
Ben	*benA*	LCH94_24330	benzoate 1,2-dioxygenase large subunit	RHA1_ro02384	78
	benB	LCH94_24335	benzoate 1,2-dioxygenase small subunit	RHA1_ro02385	75
	benC	LCH94_24340	Benzoate 1,2-dioxygenase reductase subunit	RHA1_ro02386	72
	benD	LCH94_24345	2-hydro-1,2-dihydroxybenzoate dehydrogenase	RHA1_ro02387	75
	pobA	LCH94_25050	4-hydroxybenzoate 3-monooxygenase	RHA1_ro02539	54
Van2	*vanB2*	LCH94_28925	vanillate *O*-demethylase, reductase	RHA1_ro04163	38
	vanA2	LCH94_28930	vanillate *O*-demethylase, oxygenase	RHA1_ro04165	34
Mdf2	*fmhA2*	LCH94_28940	*S*-formyl-mycothiol hydrolase	RHA1_ro02586	39
	fdhF2	LCH94_28945	formate dehydrogenase	RHA1_ro02585	84
	fdhF	LCH94_28955	formate dehydrogenase	RHA1_ro02585	77
	fdhD	LCH94_28965	formate dehydrogenase accessory protein	RHA1_ro02584	78

[a]Not included are *hpe* and *acp* genes described in the main text and Table 1
[b]Abbreviations: Pca, protocatechuate; Mdf, methionine-dependent formaldehyde, βKA, beta-ketoadipate; Van, vanillate; Cat, catechol; Phe, phenol; Gco, guaiacol; Ben, benzoate
[c]Ortholog in *Rhodococcus jostii* RHA1 used for annotation; all gene names here are the same for GD02 and RHA1; most of the orthologs in RHA1 are functionally characterized; the remainder are very similar to functionally characterized orthologs in related strains
[d]Percent amino acid sequence identity calculated over the entire length of the proteins

Table S2. Growth of strain GD02 on various organic compounds. Compounds in **bold** supported growth. Abbreviations: OD, optical density at 600nm; CFUs, colony-forming units; ND, not determined; DDVA, 2,2'-dihydroxy-3,3'-dimethoxy-5,5'-dicarboxybiphenyl; GGE, guaiacylglycerol β-guaiacyl ether. CFUs were determined in cases where other measurements were unreliable due to interference by the substrate (based on results from uninoculated controls).

Substrate	Concentration (mM)	ΔOD	Δ Total Protein (μg/ml)	ΔCFU ($x10^5$)	% Substrate Removal
**acetophenone**	1.0	0.32	83	ND	ND
**acetovanillone**	1.0	0.41	76	ND	99
**benzoate**	1.0	0.32	68	ND	99
p-coumarate	1.0	0.01	0	0	0
DDVA	0.5	0.01	0.32	0.10	2
dimethyl sulfoxide	1%	0.00	2.8	0	ND
2,6-dimethoxyphenol	0.5	0.20	1300	0	99*
4-ethylphenol	1.0	0.01	0	ND	ND
ferulate	0.5	0.03	15	8.0	38*
GGE	0.5	0.01	7	3.6	5
**guaiacol**	1.0	0.27	4.2	130	ND
**2-hydroxyacetophenone**	1.0	0.19	50	110	98
3-hydroxyacetophenone	1.0	0.02	2	0.19	16
**4-hydroxyacetophenone**	1.0	0.28	74	ND	99
**4-hydroxybenzoate**	1.0	0.26	22	ND	100
4-hydroxypropiophenone	1.0	0.00	0	1.4	0
**4-methylguaiacol**	1.0	0.20	0	610	89
**4-methylphenol**	0.5	0.13	13	120	99
4-methylsyringol	1.0	0.03	0	ND	99*
**phenol**	1.0	0.36	35	ND	ND
4-propylsyringol	1.0	0.01	0	ND	99*
4-propylguaiacol	1.0	0.01	65	0	2
syringaldehyde	0.5	0.00	0	ND	16*
syringate	0.5	0.13	0.5	ND	39*
**vanillate**	0.5	0.12	24	290	100
**vanillin**	1.0	0.25	89	ND	99

*Substrates that were also removed in uninoculated controls, thus abiologically transformed

Table S3: Components of OCF from lodgepole pine[a]

Component	OCF slurry	OCF extract
Vanillin	5.4 (0.4)	1.42 (0.01)
Vanillate	1.2 (0.1)	0.34 (0.01)
Acetovanillone	1.0 (0.1)	0.25 (0.01)
Lactate	nd[b]	0.54 (0.01)
Fumarate	nd	0.13 (0.01)
Glycolate	nd	0.64 (0.03)
2-Hydroxybutyrate[c]	nd	nd

[a]Concentration of compounds (mM) in OCF extracts totalling 2 mM aromatic compounds, based on triplicate analysis. Standard deviation is shown in parentheses.

[b]nd, not determined

[c]Predicted by comparison with the GC-MS library, not confirmed by running commercial standard.

Table S4: Oligonucleotides used to amplify *hpe* genes

**Oligo**	**Nucleotide sequence**
hpeCBA-For	TCT ACC TCA TAT GAG CAC CGA CAC CAC C
hpeCBA-Rev	TTG ACA GCT TAT CAT CGA TAA GCT TTC ACT GGG TTC CTC CGG TG
hpeD-For	AAC TTG TAT TTC CAG GGC CAT ATG AAC CGC GAC GAA CTC C
hpeD-Rev	TTG ACA GCT TAT CAT CGA TAA GCT TCT ATC CGA GCC GGG AGG G
hpeC-For	TCT ACC TCA TAT GGA AAA CTT GTA TTT CCA GGG CAT GAG CAC CGA CAC CAC C
hpeC-Rev	TTG ACA GCT TAT CAT CGA TAA GCT TTC ATG GCG TGC CTG CCT C
hpeH-For	CAA CTTT CAT ATG ACG ATC ACC GAG AGC AAG CGG AAC TC
hpeH-Rev	TGT AAG CTT CAG GAG GCG TCA GCG GCC TCG TC
hpeI-For	AAC TTG TAT TTC CAG GGC CAT ATG GGT AAG TAC ACG AAG AAG TTC AAC
hpeI-Rev	TTG ACA GCT TAT CAT CGA TAA GCT TTC ATC GGG TCT GTC CCT TC

SI References

1. Ausubel, F. M. *Short protocols in molecular biology: a compendium of methods from current protocols in molecular biology* (Fifth edition.). Wiley (2002).
2. Vaillancourt, F. H., Han, S., Fortin, P. D., Bolin, J. T. & Eltis, L. D. Molecular Basis for the Stabilization and Inhibition of 2,3-Dihydroxybiphenyl 1,2-Dioxygenase by t-Butanol. *Journal of Biological Chemistry* **273**, 34887–34895 (1998).
3. Bushnell, B. *BBMap: a fast, accurate, splice-aware aligner*. (2014).
4. Wick, R., Volkening, J. & Loman, N. Porechop. *Github https://github. com/rrwick/Porechop* (2017).
5. Wick, R. R., Judd, L. M., Gorrie, C. L. & Holt, K. E. Unicycler: Resolving bacterial genome assemblies from short and long sequencing reads. *PLOS Computational Biology* **13**, e1005595 (2017).
6. Manni, M., Berkeley, M. R., Seppey, M., Simão, F. A. & Zdobnov, E. M. BUSCO Update: Novel and Streamlined Workflows along with Broader and Deeper Phylogenetic Coverage for Scoring of Eukaryotic, Prokaryotic, and Viral Genomes. *Molecular Biology and Evolution* **38**, 4647–4654 (2021).
7. Parks, D. H., Imelfort, M., Skennerton, C. T., Hugenholtz, P. & Tyson, G. W. CheckM: assessing the quality of microbial genomes recovered from isolates, single cells, and metagenomes. *Genome Research* **25**, 1043–1055 (2015).
8. Jain, C., Rodriguez-R, L. M., Phillippy, A. M., Konstantinidis, K. T. & Aluru, S. High throughput ANI analysis of 90K prokaryotic genomes reveals clear species boundaries. *Nature Communications* **9**, 5114 (2018).
9. Edgar, R. C. MUSCLE: multiple sequence alignment with high accuracy and high throughput. *Nucleic Acids Research* **32**, 1792–1797 (2004).
10. Kumar, S., Stecher, G., Li, M., Knyaz, C. & Tamura, K. MEGA X: Molecular Evolutionary Genetics Analysis across Computing Platforms. doi:10.1093/molbev/msy096.
11. Jones, D. T., Taylor, W. R. & Thornton, J. M. *The rapid generation of mutation data matrices from protein sequences*. *CABIOS* vol. 8 https://academic.oup.com/bioinformatics/article/8/3/275/193076 (1992).
12. Fairhead, M. & Howarth, M. Site-specific biotinylation of purified proteins using BirA. *Methods in Molecular Biology* **1266**, 171–184 (2015).
13. Cornish-Bowden, A. *Analysis of enzyme kinetic data*. (1995).
14. Silverberg, L. J., Dillon, J. L. & Vemishetti, P. A simple, rapid and efficient protocol for the selective phosphorylation of phenols with dibenzyl phosphite. *Tetrahedron Letters* **37**, 771–774 (1996).
